# A cognitive behaviour data analysis on the use of social media in global south context focusing on Bangladesh

**DOI:** 10.1038/s41598-023-30125-w

**Published:** 2023-03-14

**Authors:** Shashwata Sourav Roy Samya, MD. Shaleh Islam Tonmoy, MD. Forhad Rabbi

**Affiliations:** 1 Department of Physics, Shahjalal University of Science and Technology, Sylhet, Bangladesh; 2 Department of Computer Science and Engineering, Shahjalal University of Science and Technology, Sylhet, Bangladesh

**Keywords:** Computer science, Statistics

## Abstract

This study aimed to investigate the factors that contribute to the propagation of COVID-19 vaccine misinformation on social media in Bangladesh. We attempted to identify the links between the propagation of misinformation and the factors associated with trust in sources based on personal ties among our respondents. In order to find our targeted outcomes, we used a cognitive method in our survey. A total of 202 replies were chosen for analysis, in which respondents were presented with falsified news and asked how they would react to it being shared or posted by someone with whom they have a personal connection. The survey also recorded a variety of other parameters. The Likert Chart Scale was our primary method of data collection, with Yes/No responses serving as a secondary option. The responses were analysed using statistical methods such as Chi-Square Tests, data visualizations and the Ordinal Logistic Regression Model. Our findings have shown that trust in the source can lead to hastily sharing news on social media platforms without proper verification. Other key factors, such as time spent on social media platforms and the type of content shared, also contribute to the propagation of fake news on social media platforms. Such findings may contribute to making Bangladesh more safe and secured in the cyberspace area.

## Introduction

When social media networks such as Facebook and Twitter first came in touch with third world countries, it was mainly used by people who understood it - knowledgeable enough to grasp what such platforms are actually. Internet connectivity was not something that was at one’s fingertips back then. The main obstacle of using the Internet in Bangladesh was its distribution and the device to access it. The Internet facility was still an urban privilege in Bangladesh as the telephone connections were more concentrated in urban areas, specially Dhaka—the capital of Bangladesh—based^1^. As years passed, the number of people with access to the internet increased exponentially as budget smartphones became readily available along with affordable internet packages. By the end of July 2021, Bangladesh had 123.74 million internet subscribers, almost 75% of the total population, of which 113.69 million are mobile phone subscribers^2^. Users gradually got familiar with it and thus everyone became aware of the role these platforms played in supplying information and news to the greater audience—the ease and the swift nature in which you can deliver it to the public. Such easiness prompted people to express their own views and opinions publicly on social media networks. As a result, users now possess the ability to make up news or share anything that comes up on their feed. This in turn has galvanised the spreading of fake news—“fake news” has been defined as disinformation spread through the media and then propagated through peer-to-peer communication^3^—on these social platforms^4^.

One can just look into what happened during the Road Safety Movement in Bangladesh during 2018^5^. Hundreds and thousands of students took to the streets in protest of a road accident that took the lives of two high-school students in Dhaka. The protests, the demonstrations all took place rapidly just by the click of a button on these social media platforms^6,7^. Adding fire to the fuel were unconfirmed reports about rape and assaults on students that spread from one inbox to another like lightning. Friends and peers that you had known for years, siblings and cousins were pinging each other’s Messenger and WhatsApp successively antagonising the students. These were later known to be verified as not true^8^.

Another prime example is the communal violence that is widely prevalent in Bangladesh^9^. On occasions there are reports of vandalism in top news portals which took place on the basis of word of mouth. Sometimes social accounts are hacked and used to spread fabricated news provoking another religion. Misinformation about somebody demeaning one’s belief only leads to destruction and later verification of it being untrue goes to no avail as the damage had already been done^10^.

Bangladesh is currently on the cusp of being fully vaccinated against COVID-19 and already a lot of fake news has been disseminating relating to the effectiveness of each one, misinformation about side effects and why it might even be a conspiracy from the hierarchy. These have been in circulation both nationally and globally^11,12^.

By this paper, we hope to add to the different research works carried on demystifying fake news on social media platforms. Our work looks into how someone would be affected from fake news shared by a source that is personally known and trustworthy to the reader. To delve into this, we conducted a survey among the university students of Bangladesh. According to statistics^13,14^, the age groups in 15–19, 20–29 in Bangladesh are the most populous groups that are the most active on social media platforms^15^. This age group represents the general consensus among Bangladeshi people as lower than this age group is too raw to understand the know-hows of social media platforms whilst the higher age group would be too diverse as it heavily includes veterans and professionals and people who would be less involved on social media platforms.

## Literature review

The intentional presentation of false claims as news where the claims are misleading by design is defined as fake news^16^. With the evolution of social media platforms, production of fake news^17^ and the ease and speed at which they spread and reach us is smoother than ever^18^. The main issue arises when people faced with these fake news, believe in them. There have been numerous studies as to why it is hard for people to reject fake news. One study showed that people initially consciously represent false information as true, may be because they generalize from their ordinary experiences unless it is noted^19^.

People are more prone to sharing news on social platforms that are mainly informative and promotes awareness along with status seeking and socializing^20^. As fake news mostly comes in the form of awareness type of news, this makes it to be a recipe for disaster. Even if people are able to discern which news headlines are true or false to an extent, when it comes to sharing, such accuracy had little impact. In fact, sharing intentions for false headlines were much higher than assessments of their truth^21^. Emotional factors also play an important role in sharing while political and extremist ideological views also does play its part in it^22^.

Such is the concern and the conundrum related to fake news, a vast amount of work as been done on it in a variety of ways over the past decades.

**Analyses on how to prevent fake news**

A good number of works has gone into understanding, controlling and halting fake news. People have performed a person’s behavioural analysis and looked into their personalities. Tweets from different profiles had been analysed to find what behavioural factors may contribute to the spreading of misinformation, simple binary classification models were used^23^. Linguistic patterns and personality scores using a Five-Factor Model (FFM) were also observed with a CNN Model to try to distinguish a fake news spreader from a fact checker^24^. A similar work for the problem was approached as a binary classification task and considered several groups of features, BERT semantic embedding and sentiment analysis from English and Spanish news.^25^. Analysis of verified and unverified social accounts, the number of posts and the counts of followers and following one had was also taken into consideration^26^. The predictors of Politically-slanted fake news (FN) which are usually conspiratorial in nature and often negative were examined using correlation analysis^27^.

**Investigation on the structure of fake news**

The structure of a fake news has also been investigated for detection and psychological impact it might have on an user in the past few years. Detection of fake news has been tried by using discourse segment structure analysis^28^. A similar study using hierarchical discourse level structure had also been investigated^29^. Collection of fake news via crowd sourcing and extraction of Linguistic features had also been done and run on a SVM classifier and five fold cross validation for detection.^30^. A Multi-Source Multi-Class Fake News Detection (MMFD) has been studied primarily using a CNN-LSTM model^31^. A very similar structure for MMFD with a combination of CNN and LSTM was applied but was approached by SPOT method, a fake news detection method based on semantic knowledge source and deep learning^32^. A study on the psychological appeal of a structure of a fake news has also been conducted^33^. Cognitive approaches such as changing the presentation and highlighting the source of news was also overtaken to check if the user’s belief in the article had been influenced^34^.

**Evaluation on the response towards fake news**

Further works were also completed on how a user would evaluate or respond after encountering a fake news. A qualitative research method with a descriptive approach was done among a set of university students to see how they evaluated a fake news^35^. The same had been done for a set of college students in how they identified those fabricated news along with their behavioural traits and time taken.^36^. Fake news was supplied about undergraduate students to assess their believability, credibility, and truthfulness on them upon which behavioural analysis was performed as well as neurophysiological analysis such as confirmation bias.^37^. An in depth interview study was done to see how social media users from Singapore responded to encountering fake news and results were evaluated by the pattern of their responses^38^.

**Modelling and analysing trust in fake news domain**

The trustworthiness of any source also came under scrutiny in the propagation of fake news. A study performed to see if the level of trust source had effect on the spreading of fake news^39^. A systematic review into all the studies from 2012 to 2020 to get an understanding of a user’s trustworthiness on social media networks^40^. An in depth qualitative analysis of what trust is and what is its role in information science and technology^41^. A computational trust framework for social computing where a trust value can be quantified for a piece of information^42^. Trust in sources which lead to not verifying any news and its relation to the propagation of fake news has also been heavily investigated in a number of studies^43–45^.

**Inducing emotions with fake news as stimuli**

Non algorithmic works included in looking at different tweets, classifying them, the tweet replies that may induce different emotions to the user and how novelty of the fake news contributes to its spreading^46^. Two similar works involved dealing with replies on Facebook posts. In both the works, the role of conformity of other social media users’ replies were investigated^47,48^.

Taking inspiration from these works we have gone on to work on this research paper with a non algorithmic approach to find if trust in the news source could be a big influence on the propagation of fake news. We wanted to look if our responders would skip verification if they felt the source is someone personally close or trustworthy and instantly share news on different social media platforms.

## Hypotheses

In our paper we have considered the following null hypotheses:

${\textbf {H}}_0{\textbf {1}}$: **Personal bonds do not act as a catalyst in hastily sharing a news which leads to it not properly being verified.**

We consider “personal bonds” as the relationship between someone and their parents or close family members. Respected professors, trusted friends and peers also fall in this domain for our research work. The hypothesis established here states that the personal relationship between the user and the news source is not an impulsive factor that makes the user hastily share any news on social media platforms whilst not verifying it.

${\textbf {H}}_0{\textbf {2}}$: **Concern about a particular topic (in this case Covid-19) is based less on trusting the person sharing it but more on verification of it by oneself.**

The hypothesis established here states while facing any news on social media platforms related to Covid-19 that may instill a sense of concern, the user’s worry is largely dependant on him verifying it and reconfirming the news rather than the trust the user has on the news source.

${\textbf {H}}_0{\textbf {3}}$: **People who are prone to spreading fake news on social media platforms spend a small portion of their daily internet usage on social media platforms.**

The hypothesis established here states that any user that is vulnerable in propagating fake news is interacting on social media feeds less compared to the overall amount of time he spends on the internet as a whole surfing different websites.

## Method

### Data collection

Our survey was conducted via google forms in the 1st week of April 2021. During that time the vaccination drive in Bangladesh was at its peak. The form was supplied to students through different messaging platforms and groups on Facebook. Confidentiality was managed by placing anonymous coding for each self-report questionnaire. We originally recorded 216 responses, after cleaning and removing outliers, we got a final sample of 202 students.

No humans were harmed physically or mentally while filling up the survey, the participants were mature enough to understand the questions being asked. We followed International and Shahjalal University of Science and Technology (SUST) Ethics Committee’s guidelines while gathering data from the survey. All experimental protocols were approved by Shahjalal University Human Research Ethics Committee (HREC). All participants were informed that their details would be kept anonymous, and their consent was taken before they started filling up our survey.

**Figure 1:**
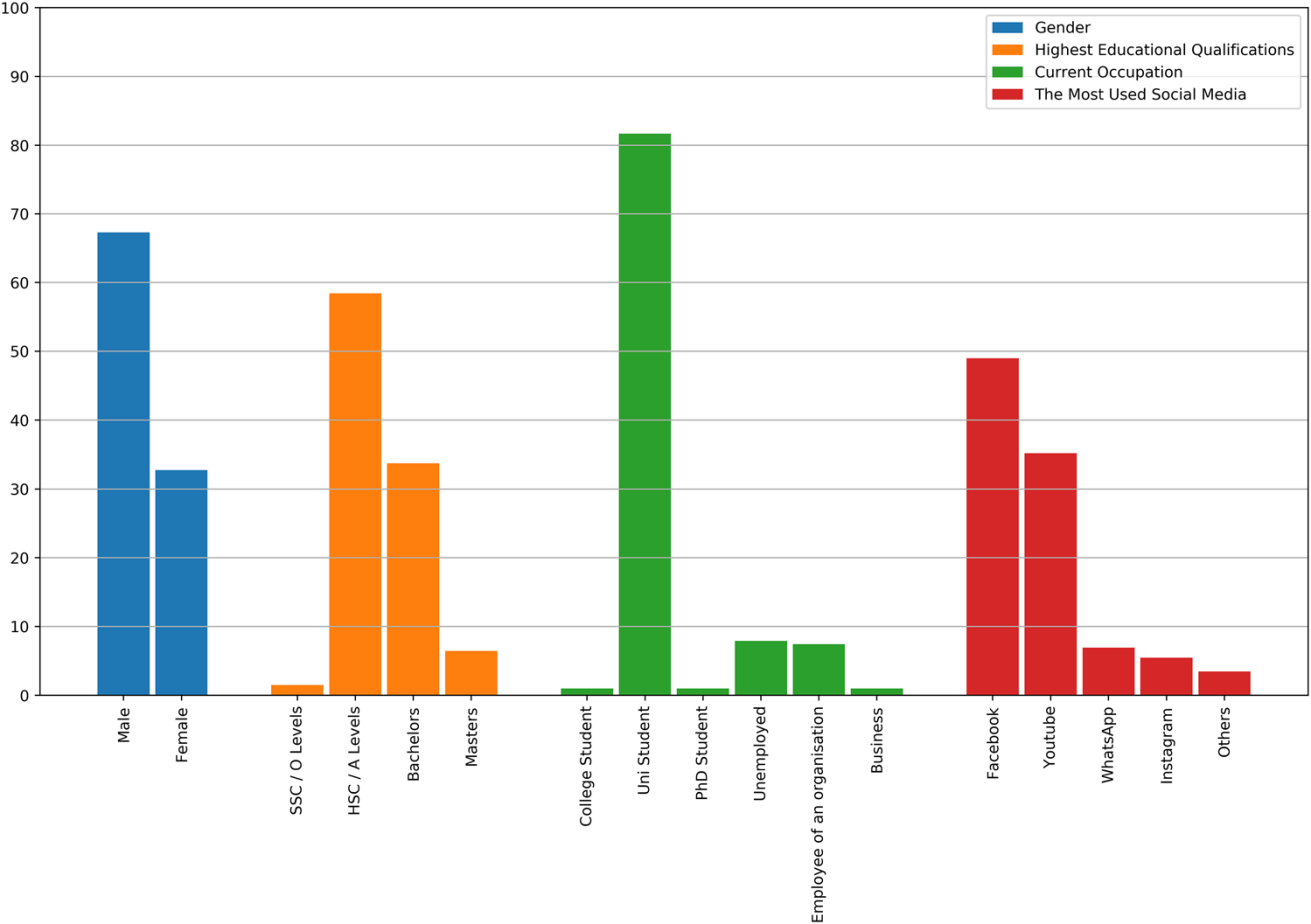
Demographic distribution.

The responders were faced with demographic questions initially which included age, gender, highest educational qualification and their current occupation status. We even asked them about their daily social media usage; how long they had been using these platforms and also their familiarity with the whole internet. The number of posts they shared and the type of posts they interacted with were also recorded. The major demographic information are depicted in terms of percentages in Fig 1.

In the immediate next part of our survey, we supplied our responders with fabricated news as stimuli about the problems related to COVID-19 vaccines displayed from usually acclaimed sources in Bangladesh and asked a series of questions related to them. All the survey takers were supplied the same fabricated news from the same sources to reduce any biasness. Questions ranged from us asking about how it made them concerned about their friends and families to how desperate they were to share these as soon as they finished the survey. The supplied fabricated news are given in the appendix A.1 as supplementary materials.

In the latter sections, they were questioned on how they would generally react to any news that would come up on their feeds and how their reactions might be if the source was from someone they trusted and admired. We even asked them if they were aware of the consequences and the outcomes of their sharing or posting. At the end of the survey, we made sure we declared in writing and pictures that the news supplied were fabricated as given in the appendix A.2.

### Measurement

Likert scales with 7 points were used in collecting these types of data ranging from strongly negative (1) to strongly positive (7). The inspiration for using a 7 pointer Likert Scale was taken from the works of Colliander^47^. Other answers were tick boxes and just a few of them were descriptive. The 9 main features that we worked on for our cognitive behaviour data analysis are as follows:

**Features measured with multiple choice options:**

*Share FNF*. This variable defined if an individual would share our fabricated news among their friends and families on social media platforms. It consisted of 3 options: “yes”, “no” and “maybe”.

*Share FNF Urgency*. This feature defined if an individual would want to share our fabricated news as soon as the survey was completed. It consisted of 3 options: “yes”, “no” and “maybe”.

*Share FNF Personal Trust*. The individual was asked if he/she would share our supplied fake news if it came on their feeds via someone they admired, respected or trusted. It consisted of 3 options: “yes”, “no” and “maybe”.

*Connected FNF*. This variable defined is an individual was connected with their friends and families on social media platforms. It consisted of a “yes” and “no” option for the surveyee to answer.

*Correct News FNF*. This feature was measured by 3 options: “yes”, “no” and “maybe”. The question asked if our surveyee would correct their friends or families if the news shared or posted by them was untrue.

*Fake News Believe*. This was a question asked at the very end of our survey to see if an individual believed in the fabricated news that was supplied to them at the beginning. It came with 2 options: “yes” and “no”.

**Features measured with a 7 point Likert Scale:**

*Urgency FNF*. This variable measured how instantly would an individual share any form of news or posts that came from someone that they trusted or respected very much.

*Trustworthy FNF*. This was a measure of how credible and reliable our surveyee found any posts or news that came from a source they personally knew or someone respectable to them.

*Important to share FNF*. This feature estimated how important an individual found to share any news that came from sources that they trusted upon.

The study of these features were not randomized in the survey. Randomization was not done because it was important for us to keep the effect of the stimuli while recording the raw responses of our survey takers for these features.

### Data preparation and statistical analysis

Initially, we cleaned our dataset by removing responses that were invalid along with the ones that were outliers. Categorical responses were encoded to numbered variables so that they were in a ranked order to aid our use of Ordinal Logistic Regression. Responses which were in the form of 7 point Likert Scale were converted into a 3 point one. This was done to get a good distribution of frequencies on both sides of the Likert Scale as some Likert Scale values yielded with very low frequencies. The conversion was done by averaging out the scales from 1 to 3 relating to their corresponding frequencies and also the scales from 5 to 7 in the same way. Scale position 4 was kept the same to use it as a neutral value. In this way, not only we got a representation of the average values of the responses for a certain feature on both sides of the scale but also it helped us to get a proper distribution of responses on the 3 point conversion that we made; scale positions 1–3 were the lower end, scale position 4 was the middle, scale positions 5–7 were the higher end.

Upon cleaning and preparing our dataset, we initially performed *Chi square tests* among the features to see if there were any significance between them. The *Spearman Rank Correlation Coefficient* was also calculated to get a better understanding of the nature of our data. Finally *Ordinal Logistic Regression* was used to see how the features related to one another. As each feature was separately tested with one another and not as a whole group, no correction for multiple testing was done in our analysis. We also explored various other *graphical methods and visualizations* to get a more in depth understanding of our dataset.

## Findings

### Relation with time

There are 3 features related to time in our work:

1. *Time Social Per Day*—the number of hours the individual spends on social media platforms
2. *Time Internet Per Day*—the number of hours the individual spends on the internet
3. *Time Internet Years*—the number of years that the individual has been using the internet for.

By the help of distribution plots we present the following findings of the main features:

**Figure 2:**
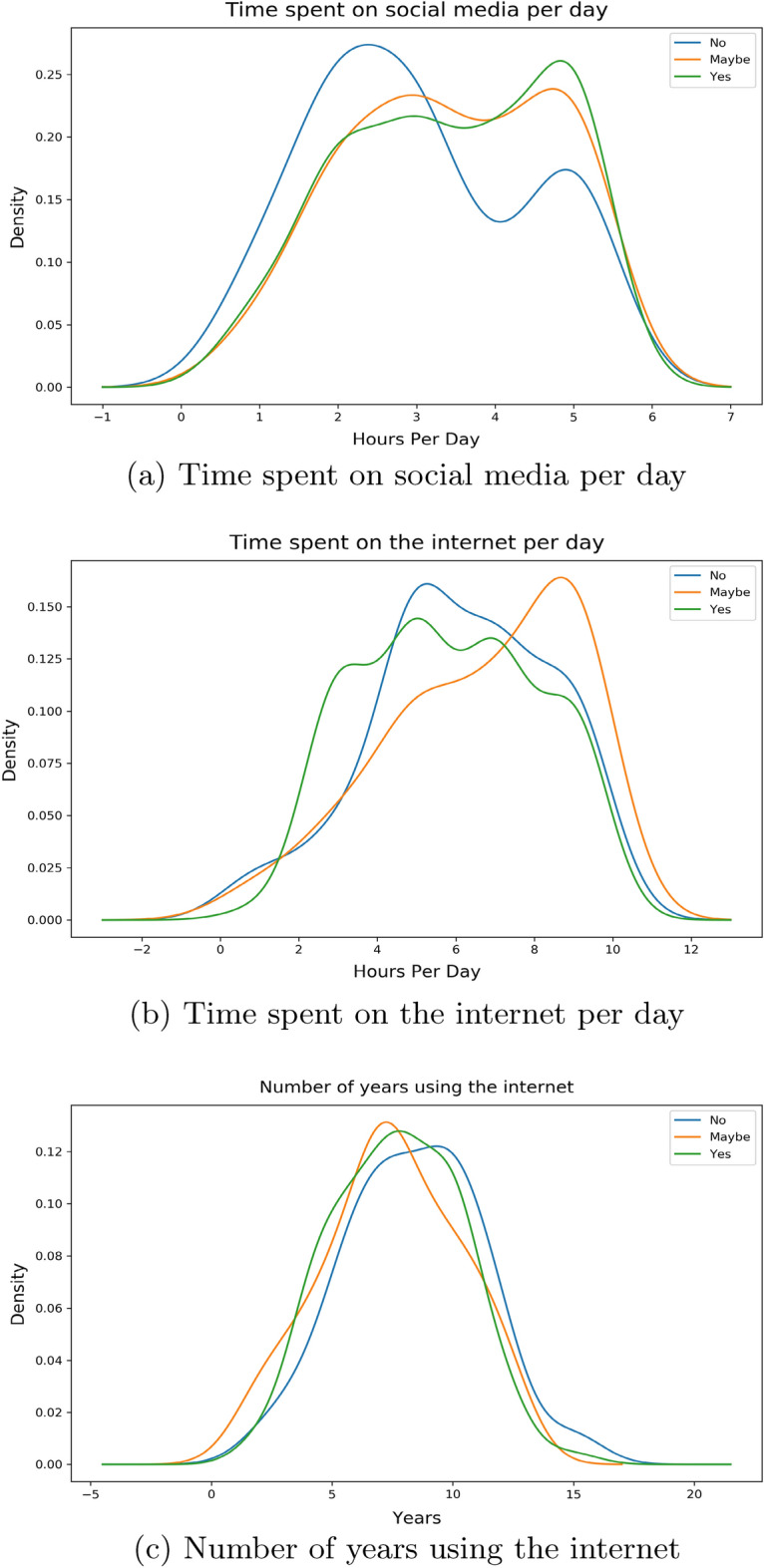
*Share FNF* distribution with time.

Here, in Fig. 2a, we can see that for an individual who would want to share our provided false news, most of them spend around 5 h per day on social media compared to their counterparts where the majority spend just over 2 hours on these platforms. If we look into Fig. 2b, we see that the surveyees who would and wouldn’t want to share peak around the same part of the graph whereas people with “maybe” as their option spend more time on the internet compared to them. The number of years using the internet for these people are uniformly distributed in a similar way as shown in Fig. 2c.

**Figure 3:**
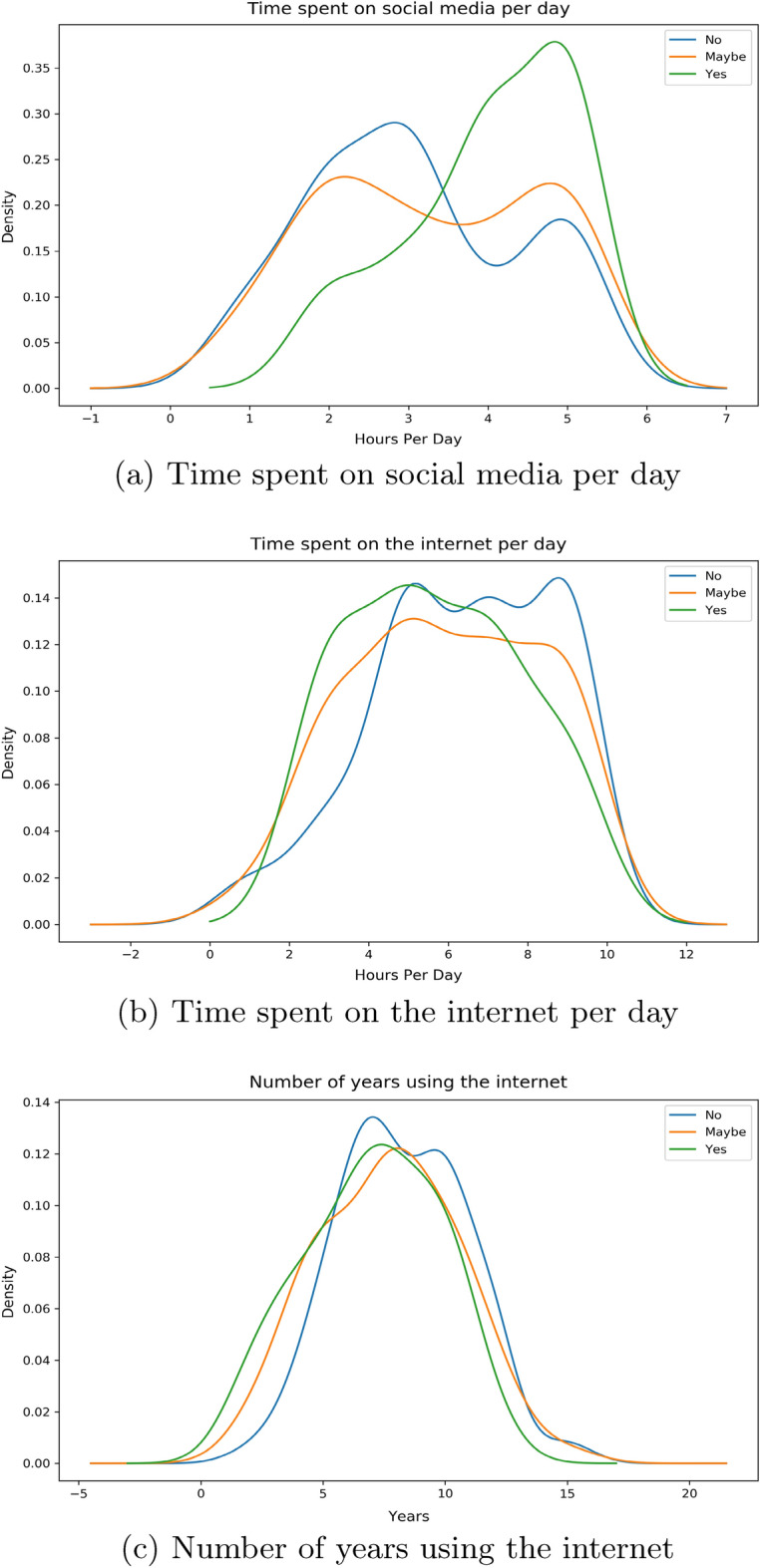
*Share FNF Urgency* distribution with time.

For this feature we observed that there is a much bigger spike for an individual who would urgently want to share our supplied fabricated news among peers around the 5 h mark according to Fig. 3a. For the group of individuals who do not want to share as soon as finishing our survey, most of them are spending around 2–3 h per day. Moving over to the time spent on the internet per day in Fig. 3b, we see people with “yes” as their answer spending slightly less time on the internet on average compared to those who answered “no”. The number of years using the internet distribution is again found to be distributed to in a similar way in Fig. 3c.

**Figure 4:**
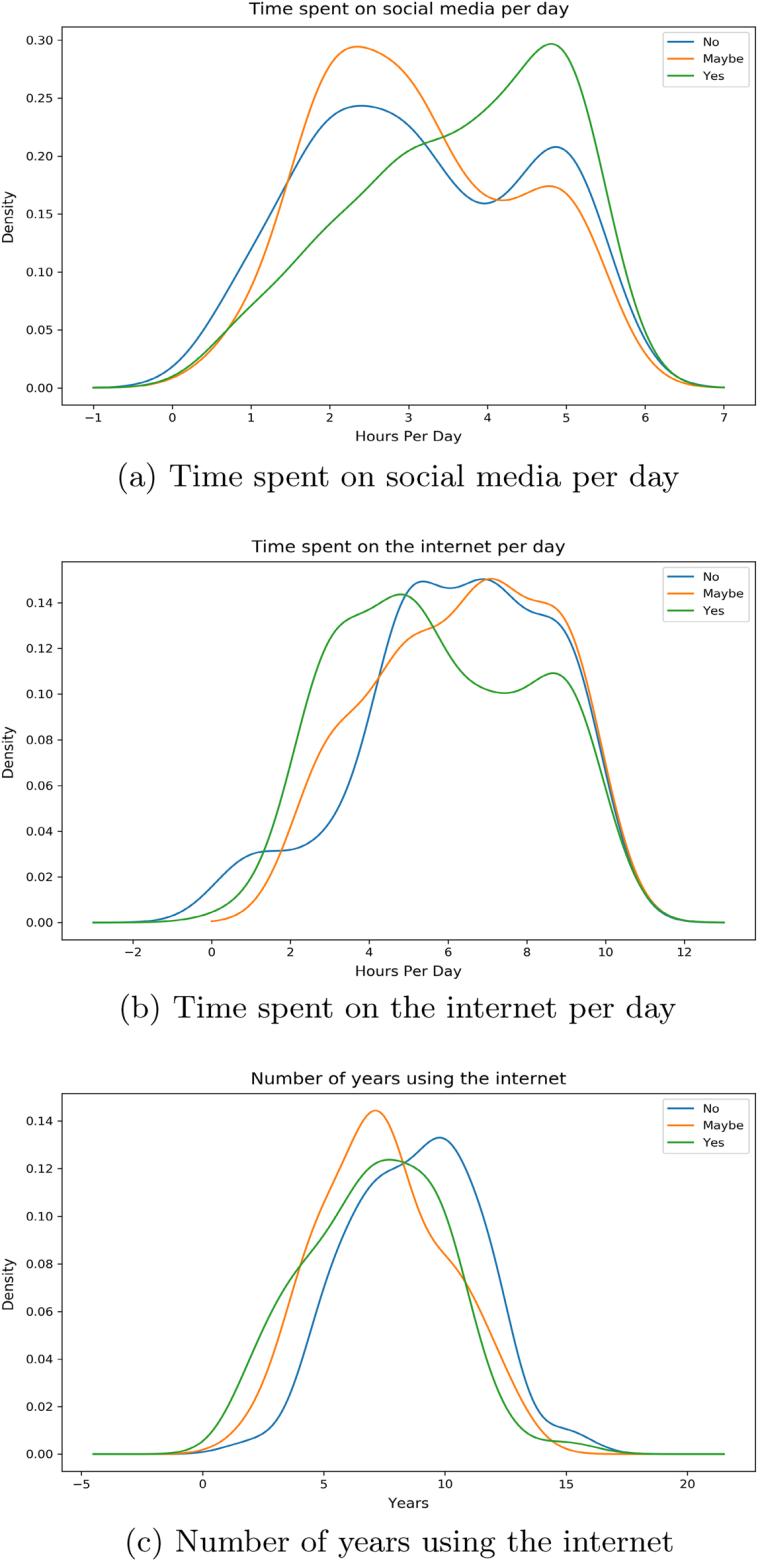
*Share FNF Personal Trust* distribution with Time.

From our Fig. 4a we can observe a similar distribution much like our previous two features. In terms of the group of individuals with “yes”, a large portion of them expend around 5 h compared to their counterparts who spend around 2 h. Majority of the responders with “maybe” are seen to share a similar peak but only higher like the responders who answered “no”. Moving on to Fig. 4b, we notice it following a similar pattern to our previous features. Individuals answering “yes” are exhausting around 4 hours whereas individuals with “yes” are doing so for 5–8 h. A uniform distribution is observed for the number of years in using the internet in Fig. 4c. “Yes” group of people have been around the internet for slightly less years compared to people with “no”.

**Figure 5:**
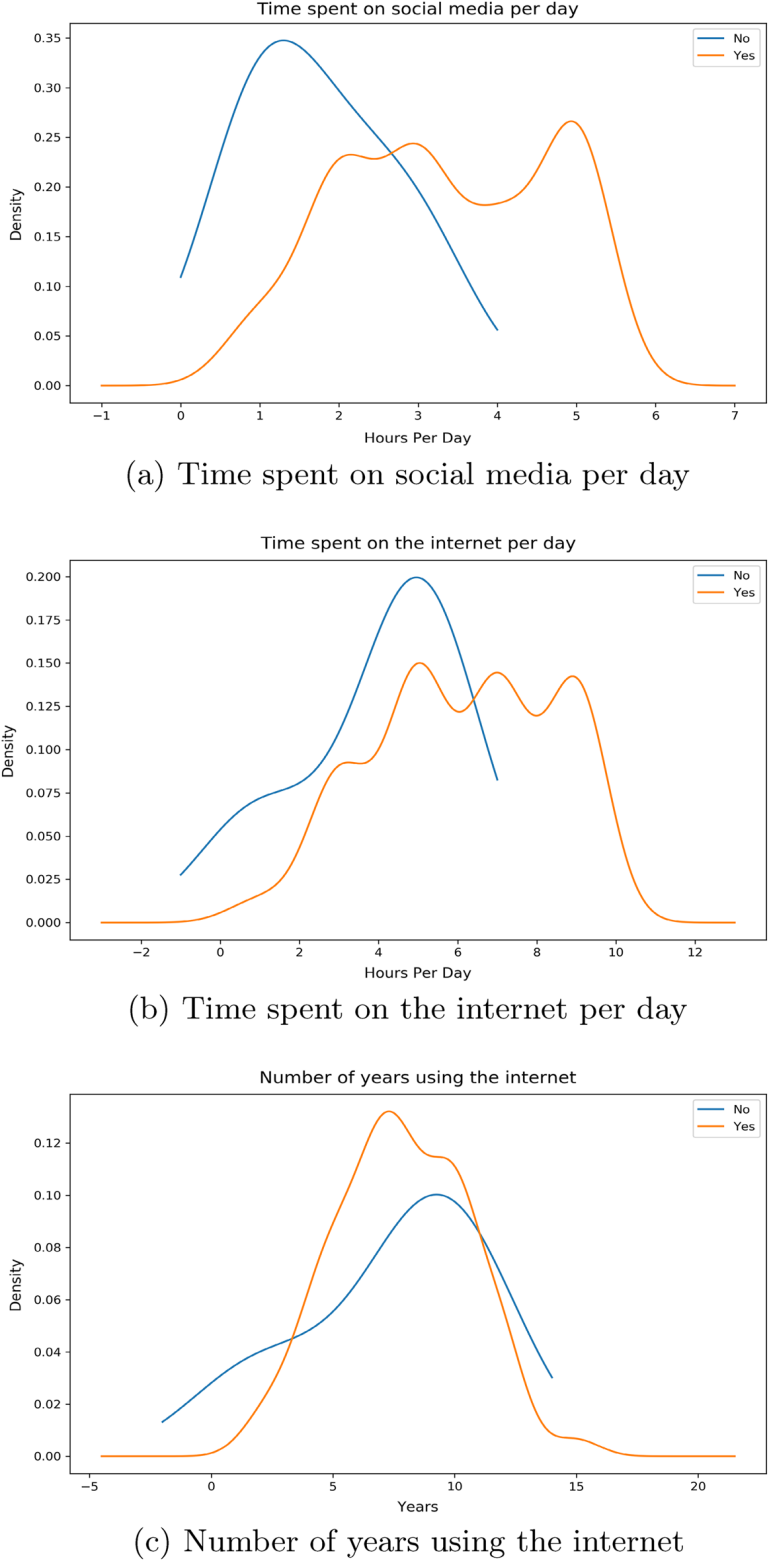
*Connected FNF* distribution with Time.

From Fig. 5a, we observe people who are usually connected with their close ones on social media platforms tend to spend more time on social media platforms per day compared to people who aren’t. Figure 5b is showing that people who are connected with friends and family are spending time from 5 to 10 h on a the internet whereas for their counterparts we see that there is a major spike around 4-6 hours. Figure 5c is showing a similar distribution nature for both these cluster groups.

**Figure 6:**
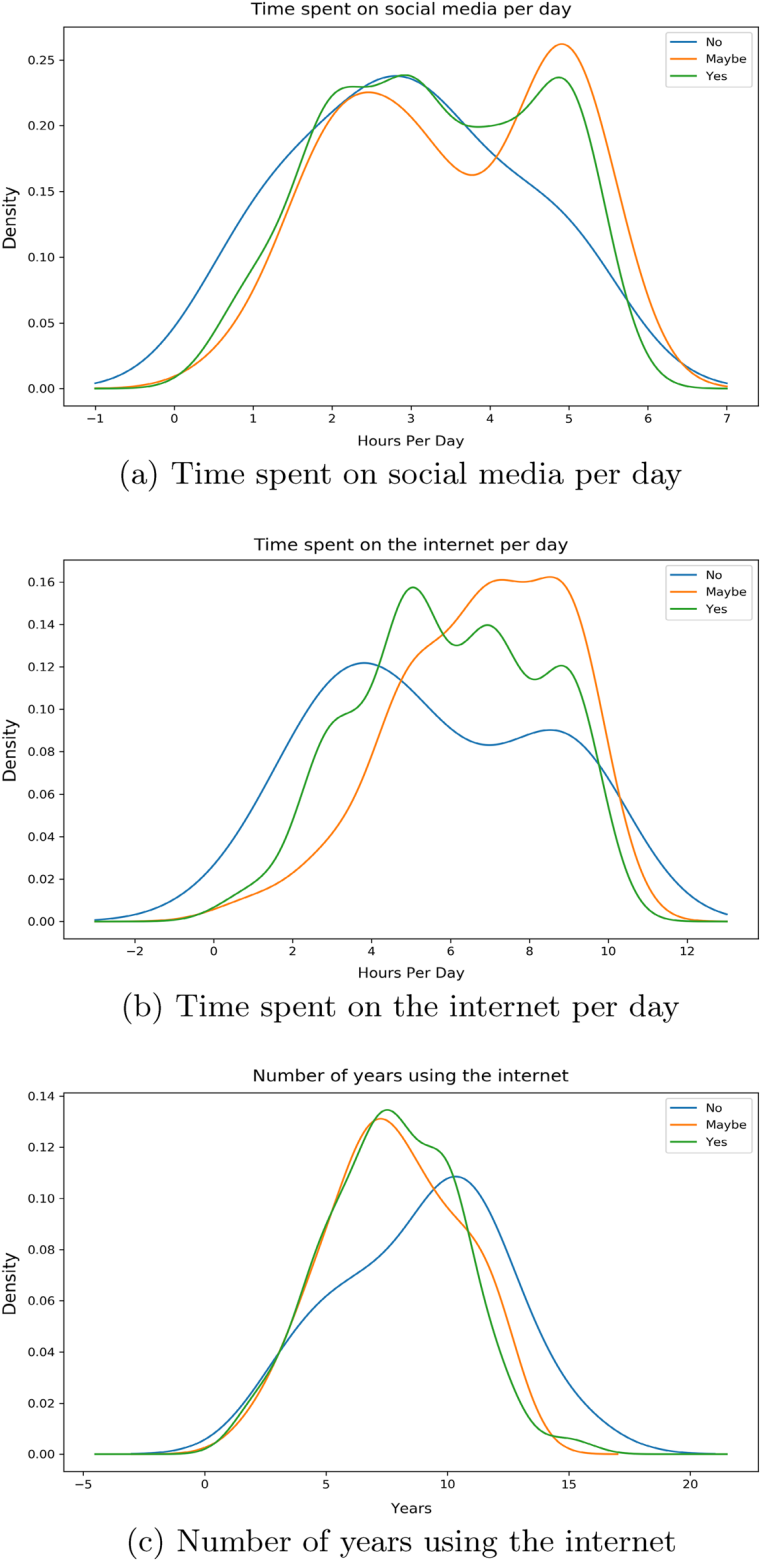
*Correct News FNF* distribution with Time.

We notice from Fig. 6a, a downward trend for people who would not want to correct their close ones if they mistakenly shared a false news from around 3 h. We see the opposite for people who would move for a correction as rising spike is seen at around 5 h. Similar trend is observed for people with “maybe” as their answer. For Fig. 6b, we detect a similar distribution over hours among both positive and negative individuals whilst the neutral individuals are showing a peak around 7–10 h. In the case for the number of years in using the internet, we see a slight deviation for individuals who responded with “no” for correction in Fig. 6c. This is portraying that people with more experience with the internet would not want to correct false news.

**Figure 7:**
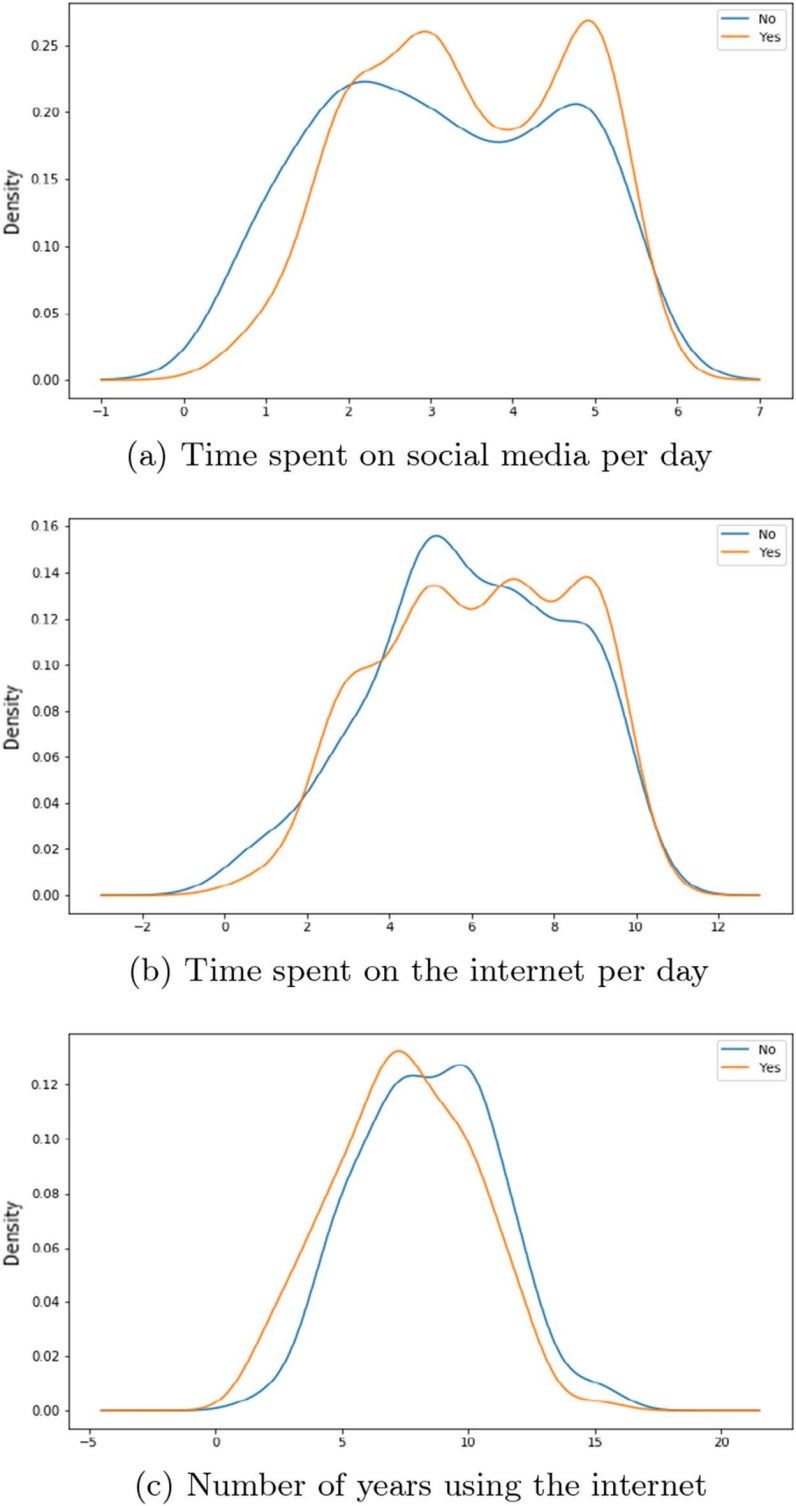
*Fake News Believe* distribution with Time.

The distribution in Fig. 7a is showing a similar pattern for both the ’yes’ and ’no’ groups. However, the density for the “yes” group is much higher in the same regions as where the “no” group has shown its peaks. This shows that there is a larger faction of responders who believed in our supplied fabricated news compared to people who did not, even though the use of social media per day was similar. Fig.7b depicts individuals who did not believe, spending a bit less time on the internet compared to their opposites. This can be understood by the highest peaks occurring on two separate sides of their middle intersection. The number of years in using the internet distribution in Fig. 7c shows both peaks are somewhat similar but the peak for “yes” is a bit earlier than for “no”, showing people with more years of internet years are comparatively better at identifying what may be fake news.

The numbers denoted in the legends of the distributions below are showing the mean value of the features on either side of the Likert Scale calculated with respect to their corresponding frequencies.

**Figure 8:**
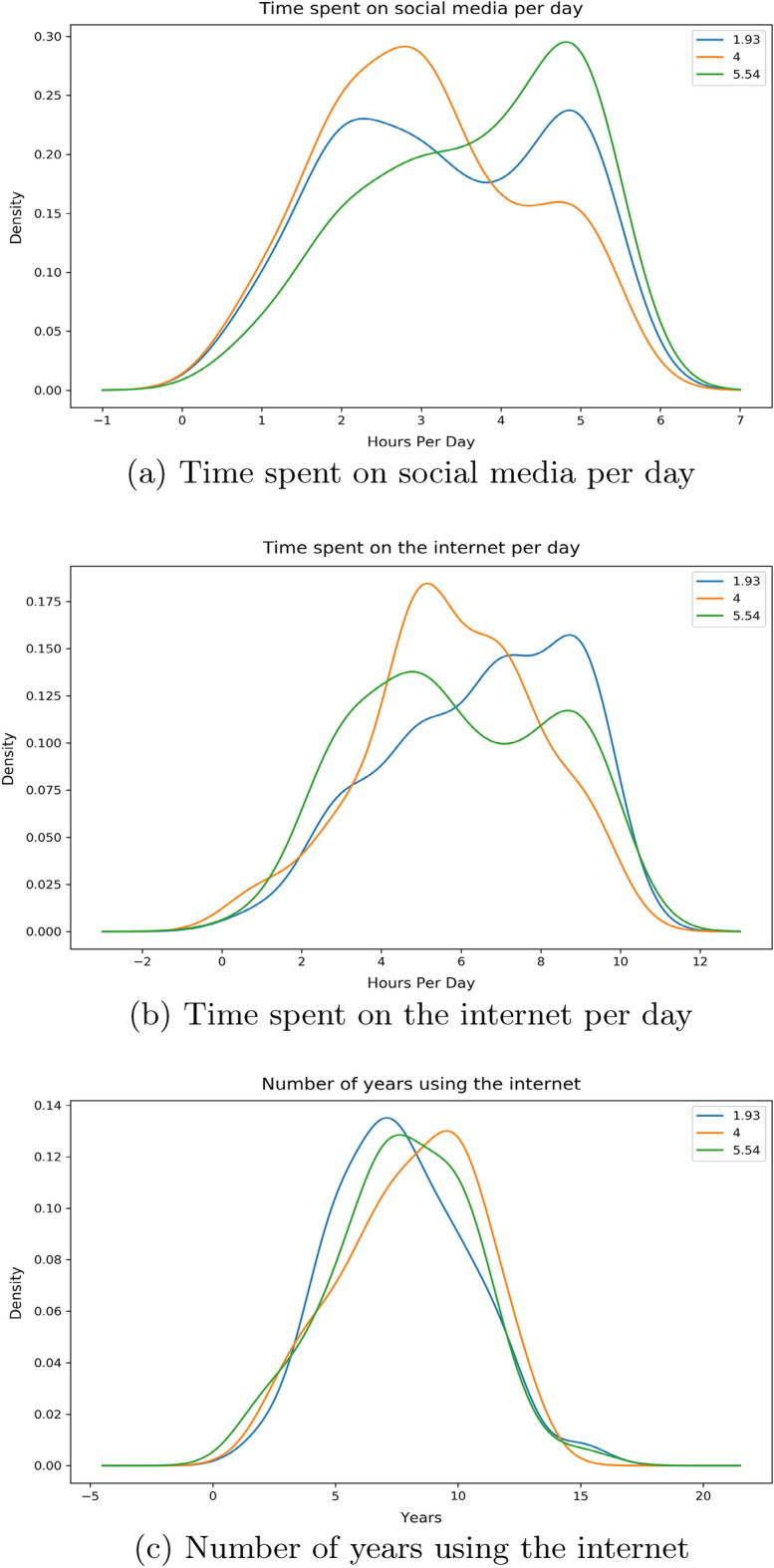
*Urgency FNF* distribution with Time.

From Fig. 8a, we observe two contrasting separate peaks of similar densities for individuals who find it urgent enough at both low and high ends whereas for individuals who are in the moderate section, a peak is seen in the lesser portion of hours per day on social media. People who find it more urgent are showing considerably more use of social media per day compared to people who find it less urgent. From Fig. 8b, we see two distinct peaks opposite to one another; individuals who find it more urgent spend less time on the internet compared to individuals who find it less urgent spending more time on the internet. The peak for the moderate section of urgency individuals is approximately between these two. The distribution for the number of years are seen to be similar for all 3 sections of the individuals as seen on Fig. 8c.

**Figure 9:**
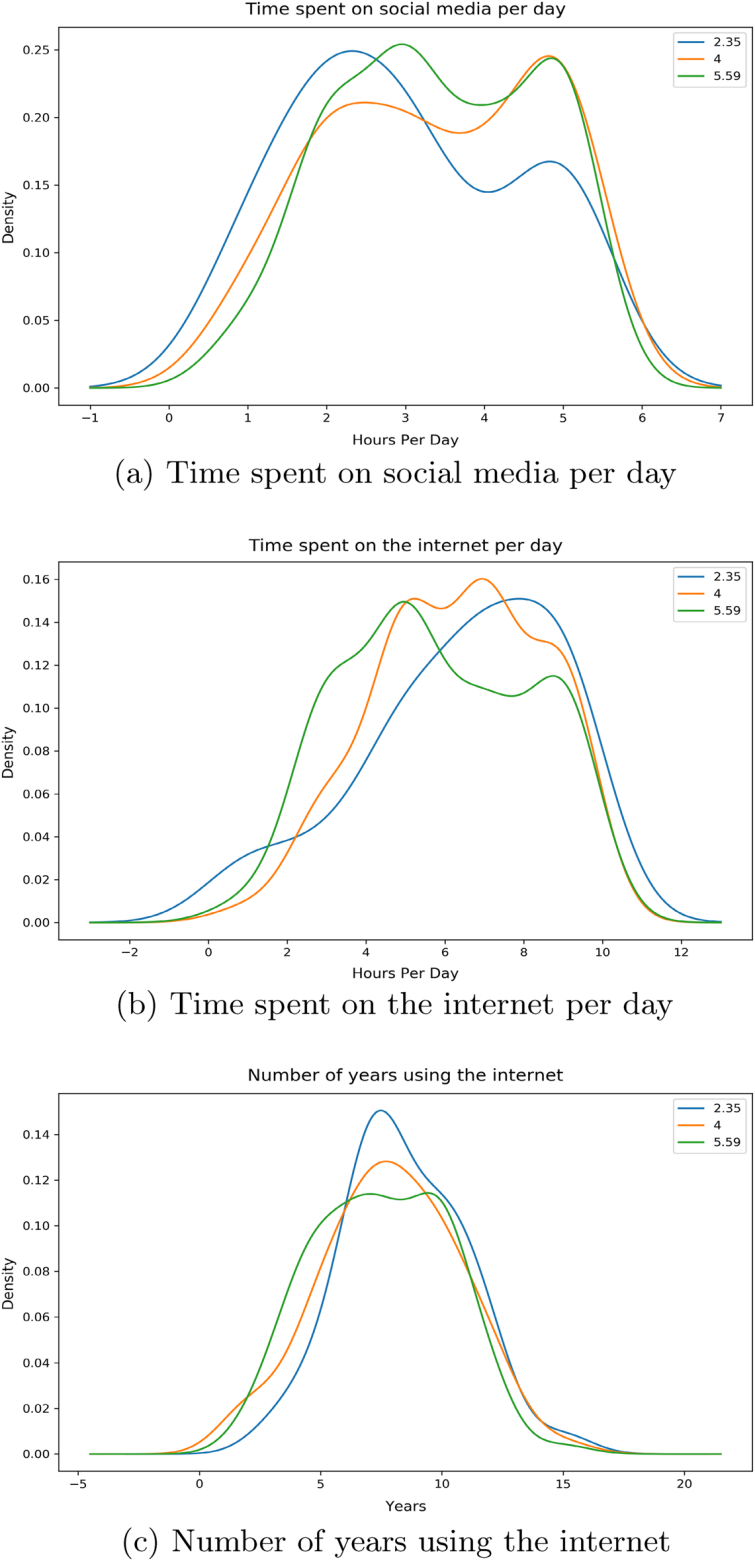
*Trustworthy FNF* distribution with Time.

For this feature, from our Fig. 9a it can be seen that people who find news less credible from personally known sources are spending relatively less time on social media platforms per day compared to people who find these news credible. The moderate section follows a similar nature to people who find it more credible. Opposite distinct peaks can also be observed for people who find it credible and who do not in Fig. 9b. People who find news and information from those personally known sources tend to exhaust less time on the internet at a peak of around 4 h compared to their counterparts at around 6 h. The moderate section is in-between both these peaks. Figure 9c shows a similar distribution for all 3 sections of the responders with people not finding news credible showing a narrow peak compared to the more spread out, blunt peak for people who find it credible.

**Figure 10:**
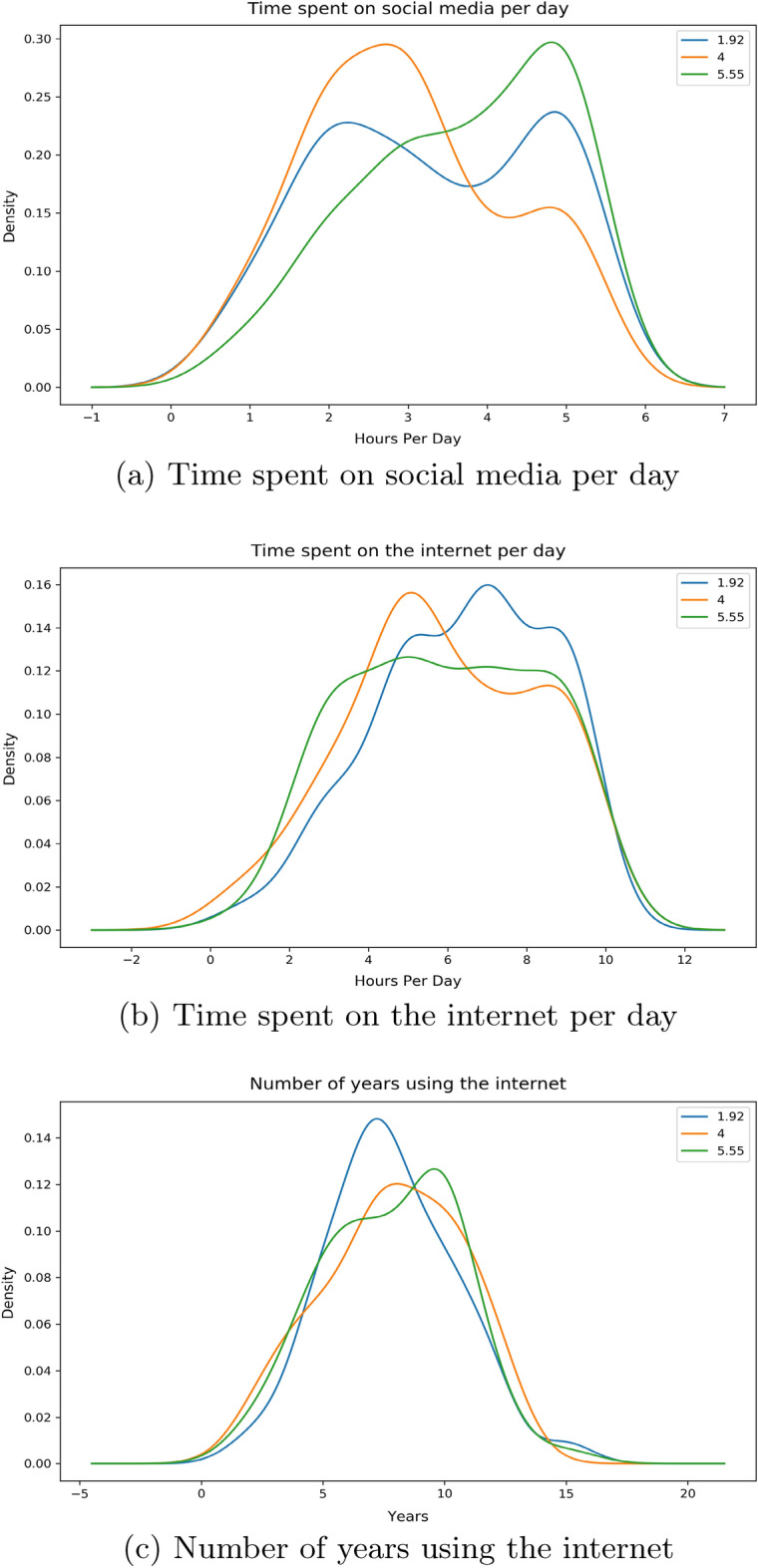
*Important to share FNF* distribution with Time.

The distribution for this feature has shown that people who find it more important to share any news from their known sources such as families and friends are spending more time on social media platforms per day with a peak at around 5 h compared to their counterparts whose times are distributed from 2 to 6 h from Fig. 10a. The moderate section is showing less time spent on social media platforms per day with a peak at 2–3 h. Figure 10b is displaying people on the lower importance spectrum spending more time on the internet with a peak at around 7 h. Individuals who find it more important are almost evenly distributed out in terms of hours spent on the internet daily. The moderate section is showing a peak at just over 4 h. From Fig. 10c we again notice a similar distribution much like our previous features for all 3 sections of groups of individuals.

### Relation between features

To find relationships between two features we have used the approach of Ordinal Logistic Regression as our responses were in ranked order and also taken a measure of their coefficient and their significance to understand the likelihood.

**New features that are included in this table are described as follows:**

*Urgency*. This variable is a measure of how hastily would the individual share the news or information coming from any source. This was measured using a Likert Scale.

*Trust Source*. This variable is a measure of how trustworthy an individual found the sources we used to show our fabricated news from. This was measured using a Likert Scale.

*Important To Let Others Know*. We asked the surveyees how important they found a news or information to let others know about using a Likert Scale.

*Motivated to Share*. The variable was used to measured how motivated one felt to share a certain news. The measuring tool was a Likert Scale.

*Consequences Aware*. This was a question with options “yes”, “maybe” and “no” to see if while sharing a news, the surveyee was aware of the consequences it may have.

*Outcome*. This was a measure of how positive or negative outcome an individual expected after sharing a news or piece of information on social media platforms. This was measured using a Likert Scale.

*After Covid Vax*. Here we asked the responders if they would take the vaccine after reading our fabricated news. It consisted of “yes”, “maybe” and “no” options.

*Elder Concern*. We asked if our fabricated news made them concerned about their elders in taking the vaccine. Consisted of “yes”, “maybe” and “no” options.

Below is a table, Table 1, showcasing the coefficients and the p-value between the features after Ordinal Logistic Regression has been performed. Only the noteworthy relations are mentioned in the table.

**Table 1:** The coefficients and the p-value among features.

Feature 1	Feature 2	Coefficient	$\hbox {P}> {\textbf {z}}$
Share FNF	Share FNF Personal Trust	1.7135	0.000
Share FNF	Share FNF Urgency	2.1452	0.000
Share FNF	Fake News Believe	0.6855	0.011
Share FNF	Urgency	0.2208	0.012
Share FNF	Trust Source	0.3721	0.000
Share FNF	After Covid Vax	-0.672	0.001
Share FNF	Elder Concern	0.8310	0.000
Share FNF Urgency	Share FNF Personal Trust	1.9624	0.000
Share FNF Urgency	Trust Source	0.2805	0.008
Share FNF Urgency	Urgency	0.2712	0.003
Share FNF Urgency	Elder Concern	0.4360	0.006
Share FNF Personal Trust	Fake News Believe	0.8093	0.003
Share FNF Personal Trust	Trust Source	0.2572	0.012
Share FNF Personal Trust	Elder Concern	0.6957	0.000
Urgency FNF	Trust Source	0.4137	0.000
Urgency FNF	Important To Let Others Know	0.5720	0.000
Urgency FNF	Motivated To Share	0.5879	0.000
Urgency FNF	Outcome	0.5682	0.000
Urgency FNF	Share FNF	0.3465	0.024
Urgency FNF	Share FNF Personal Trust	0.6085	0.000
Urgency FNF	Consequences Aware	0.8720	0.001
Urgency FNF	After Covid Vax	-0.4299	0.010
Trustworthy FNF	Trust Source	0.5244	0.000
Trustworthy FNF	Share FNF	0.4758	0.003
Trustworthy FNF	Urgency FNF	0.8564	0.000
Trustworthy FNF	Important To Let Others Know	0.6266	0.000
Trustworthy FNF	Outcome	0.6274	0.000
Important To Share FNF	Trustworthy FNF	0.9948	0.000
Important To Share FNF	Urgency FNF	1.5725	0.000
Important To Share FNF	Share FNF Urgency	0.3682	0.031
Important To Share FNF	Outcome	0.4714	0.000
Important To Share FNF	Trust Source	0.3334	0.001
Important To Share FNF	Share Personal Trust FNF	0.5450	0.000

It is important to note that any features with *FNF* in them relates to a question asked on the basis if the news is coming from a trusted source. Any features without *FNF* in them were questions asked on the basis that the news is coming from any source.

### Research involving human participants

A total of 216 human participants took part in answering our survey for this research.

### Informed consent

All participants agreed to give their consent before taking our survey.

## Results and discussion

### Related to time

Given from our distribution plots, we have been able to deduce the following from our observations:

1. When asked about features related to the fabricated news that we had supplied, the answers have shown us that individuals who wanted to inform our news to their friends and families as soon as finishing our survey spend more time on social media platforms and less time on the internet per day compared to their counterparts. The same can be said even if the news came from sources that they have trust or respect upon. Thus we believe individuals in this group who would want to urgently share news from a personally trusted human source without verification, use maximum of their time on the internet on social media platforms. Their opposite group has shown that a small to moderate chunk of the amount of time spent on the internet is exhausted on social media platforms. We have also noticed a very similar pattern in time consumption when we asked the surveyees on how they would react to any news or information being shared from a personally known or admired source in general.
  These observations can be distinctively seen with the features *Share FNF*, *Share FNF Urgency*, *Share FNF Personal Trust* and *Urgency FNF*. This pattern is also similarly seen with *Trustworthy FNF* and *Important to Share FNF*. Thus it allows us to reject our ${\textbf {H}}_{0}{} {\textbf {3}}$ and we can conclude that people who are prone to spreading fake news on social media platforms spend a large chunk of their daily internet usage on social media platforms in Bangladesh.
2. People connected with their loved ones and friends dedicate more time on social media platforms and overall on the internet on a daily basis in Bangladesh. This can be seen from the distribution of the feature *Connected FNF*.
3. In Bangladesh, individuals who feel they should let their source know if the news is false and needs to be corrected or unsure to inform, spend more time on social media over around 5 hours compared to individuals who are uninterested in correcting or letting them know. We also see the former group having low years of experience with the internet compared to the latter group. This can be seen from the distribution of the feature *Correct News FNF*.
4. Most people who believed in our stimuli have shown a higher daily usage of social media and the internet. Though we suspect a small group of individuals may have had certain bias in answering this specific question due to it being asked directly. This can be seen from distribution from of *Fake News Believe FNF*.

### Related to other features

1. From our table we have observed that *Share FNF* with *Share Personal Trust* has a coefficient of 1.7135, *Share FNF* with *Share FNF Urgency* has 2.1452, *Share FNF Urgency* with *Share FNF Personal Trust* has 1.9624 and *Important To Share FNF* with *Urgency FNF* has 1.5725. This shows if the source is personally known or trusted, the urgency in sharing is significantly more likely which in turn may lead to not verifying the news. This finding primarily rejects our ${\textbf {H}}_0{\textbf {1}}$. This observation has been made possible by introducing a fabricated news in our survey based on which our survey takers responded. If we take a closer look into the following relations of *Share FNF* with *Urgency* (Coefficient of 0.2208), *Urgency FNF* with *Share FNF Personal Trust* (Coefficient of 0.6085) and *Urgency FNF* with *Share FNF* (Coefficient of 0.3465), we end up noticing that the low coefficients are showing due to the general nature of the questions asked in those sections— the section where we asked the survey taker to record their responses according to their usual day to day activity cases on social media news interactions. This contradicts our findings for the features related to the stimuli that we introduced—the coefficients spike up as mentioned before. We believe these to be the true responses of our survey takers.
2. Looking into relations of *Trust Source* with various features such as *Share FNF* (Coefficient of 0.3721),*Share FNF Urgency* (Coefficient of 0.2805), *Share FNF Personal Trust* (Coefficient of 0.2572) and *Important To Share FNF* (Coefficient of 0.3334) further rejects our ${\textbf {H}}_{0}{} {\textbf {1}}$ and ${\textbf {H}}_{0}{} {\textbf {2}}$. The low coefficients infer to the reader having a lower trust on different news portals, but if the same news is backed up by a source that the individual personally knows, the credibility of the news that has been shared to them increases as noticed before with the higher coefficients. These results are consistent with the findings of Talwar 2019^44^, Talwar 2020^45^ and Laato 2020^43^. Talwar 2019^44^ have shown that “Online Trust” has positive relation to “Sharing Fake News Online” while has a negative relationship with “Authenticating News Before Sharing Online”. Laato 2020^43^ has shown a similar result with “Online Information Trust” having a positive relationship with “Unverified Information Sharing”. To further consolidate our findings, Talwar 2020^45^ has shown that “Instantaneous Sharing of News for Creating Awareness” having a positive relationship with “Sharing Fake News Due to Lack of Time”.
3. We have also noticed that an individual sharing a piece of information from a trusted source generally expect a positive outcome. This can be backed up by the following relations of *Outcome* with *Urgency FNF* (Coefficient of 0.5682), *Trustworthy FNF* (Coefficient of 0.6274) and *Important To Share FNF* (Coefficient of 0.4714).
4. Good amount of likelihood has been observed for Covid 19 vaccine related features in our work. *After Covid Vax* with *Share FNF* (Coefficient of -0.672) and *Urgency FNF* (Coefficient of -0.4299) does show that the individual was affected by our fabricated news and hence the likelihood of sharing the concerned news urgently or hastily is greater. *Elder Concern* with *Share FNF* (Coefficient of 0.8310), *Share FNF Personal Trust* (Coefficient of 0.6957) and *Share FNF Urgency* (Coefficient of 0.4360) further allows us to reject our ${\textbf {H}}_{0}{} {\textbf {2}}$. When a concerning news is shared or posted by someone personally trusted, the reader is more likely to share it quickly without verification as it affects them. Referencing to Laato 2020^43^, their findings showed that the worry for personal health does not lead to propagating that news further. However, we have dealt with the worry for family members and closed ones in our work and with reference to Weismueller 2022^22^, their work states emotionality is more important than argument quality in fostering sharing which solidifies our result in having emotional ties and concern which lead to sharing news unverified.

## Dataset observations

### Relation with different share types

In our survey we asked our responders to mark the different type of contents that they share or post on their social media platforms. The list contained *Awareness*, *Educational*, *Entertainment*, *Informative*, *Motivational*, *News*, *Personal*, *Philosophical*, *Political*, *Religious*, *Scientific*, *Sports* and *Others*. When compared with our 9 selected features, we have been able to make the following observations, all units are in percentages:

**Figure 11:**
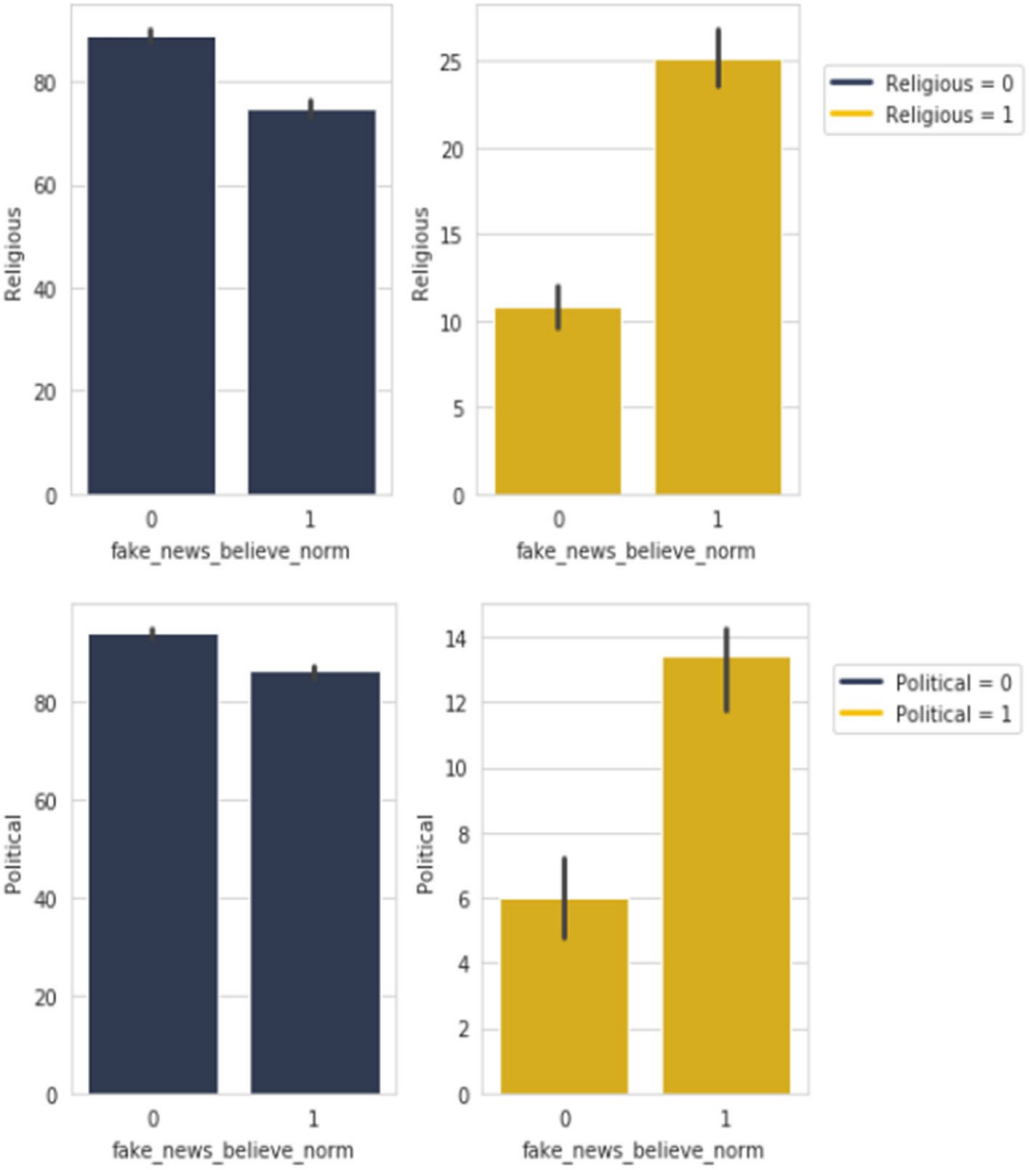
*Fake News Believe* with *Religious* and *Political types*.

It can see seen from Figure 11 that in the case of sharing *Religious* and *Political* contents, there are a higher portion of responders who believed in our supplied fabricated news compared to people who did not. The opposite is observed among those who do not share contents in this categories.

**Table 2:** Percentage of people in the positive spectrum of *Share FNF Personal Trust* and *Trustworthy FNF* who are *Fake News Believe* = 1 and share *Religious* and *Political* content.

Fake News Believe = 1			
Share FNF Personal Trust			
	Maybe	Yes	Cumulative
Religious = 1	16.7%	53.3%	70.0%
Political = 1	18.8%	50.0%	68.8%

Trustworthy FNF			
	4.00	5.59	Cumulative
Religious = 1	36.7%	43.3%	80.0%
Political = 1	18.8%	56.3%	75.1%

Looking further into how this relationship may be aligned to features relating to personal bonds, *Share FNF Personal Trust* and *Trustworthy FNF*, we can look in to Table 2. In this table we are considering the group of people who believed in the fake news we supplied (*Fake News Believe = 1*) in the survey and share *Religious* and *Political* content.

The table shows that 53.3% of these people would share if our fabricated news came from a personally trusted human source. The cumulative percentage including the “Maybe” group shows 70% of these people would share. Similar result is observed in the case of believing any kind of news coming up in social news feeds from personally trusted human source. People who would believe any kind of news from a trusted source are 43.3% with the cumulative in 80%. All these people are in the principal group of people who believed in the fabricated news we supplied which was negative, that means they did not verify if the news was untrue. This finding is consistent to the work of Weismueller 2022^22^ where its stated that ideologically extreme users are less likely to share positive content and fake news are generally considered as negative news.

**Figure 12:**
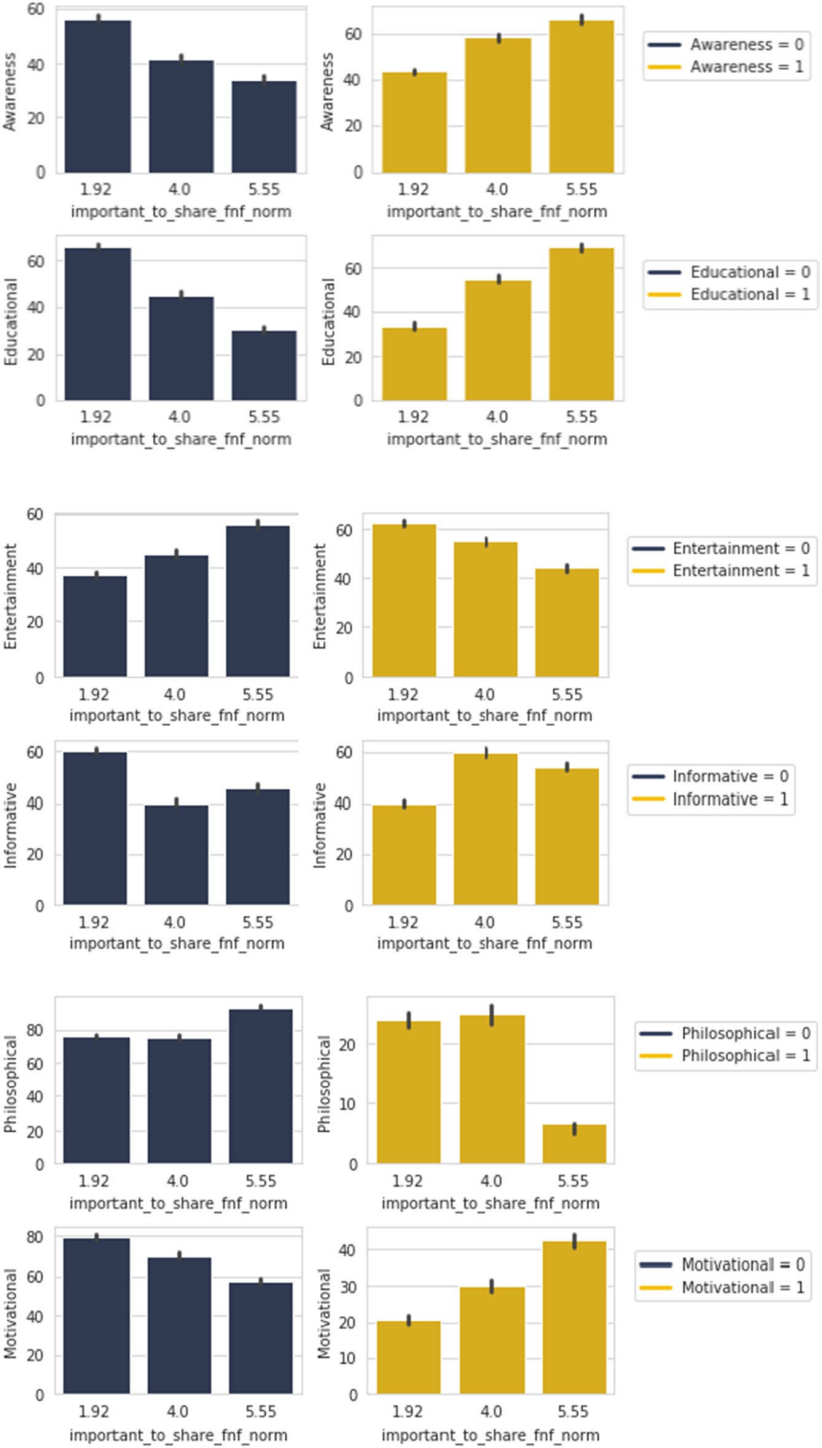
*Important to share FNF* with *Awareness, Educational, Entertainment, Informative, Motivational* and *Philosophical*.

**Table 3:** Percentage of people in the positive spectrum of *Share FNF Personal Trust* and *Trustworthy FNF* who are *Important to share FNF = 5.55* and share *Awareness, Educational, Entertainment, Informative, Motivational* and *Philosophical* content.

Important to share FNF = 5.55			
Share FNF Personal Trust			
	Maybe	Yes	Cumulative
Awareness = 1	23.1%	56.4%	79.5%
Educational = 1	29.3%	58.5%	87.8%
Entertainment = 1	26.9%	57.7%	84.6%
Informative = 1	28.1%	53.1%	81.2%
Motivational = 1	18.8%	81.2%	100%
Philosophical = 1	50%	50%	100%

Trustworthy FNF			
	4.00	5.59	Cumulative
Awareness = 1	17.9%	79.5%	97.4%
Educational = 1	22.0%	75.6%	97.6%
Entertainment = 1	23.0%	77.0%	100%
Informative = 1	18.8%	81.2%	96%
Motivational = 1	20.0%	76.0%	100%
Philosophical = 1	0.00%	100%	100%

Here in Fig. 12, contents that are shared in the category of *Awareness*, *Educational* and *Motivational* are showing an upward trend for responders who find those important to share. However, in the case of *Entertainment* and to an extent *Philosophical*, we can observe that its the opposite trend. For *Informative*, the importance in sharing is neutral. The absolute opposite nature is more or less observed among people who do not share these types in the case of importance in sharing.

Looking further into how this relationship may be aligned to features relating to personal bonds, *Share FNF Personal Trust* and *Trustworthy FNF*, we can look in to Table 3. In this table we are considering the group of people who find it important to share content from personally trusted human sources *Important to share FNF = 5.55* and share *Awareness, Educational, Entertainment, Informative, Motivational* and *Philosophical* content.

The table shows all of these share types to have percentages over 50% in terms of “Yes” and a cumulative of around 80% in all cases for people who would want to share our fabricated news if it came from a personally trusted human source. High percentages of around 80% is seen in terms of “Yes” and cumulative of over 95% for people who would believe in any news shared by trusted sources. This shows that if any news comes from a personally trusted human source on news feeds, the people who find it always important to share would believe it and share it.

**Figure 13:**
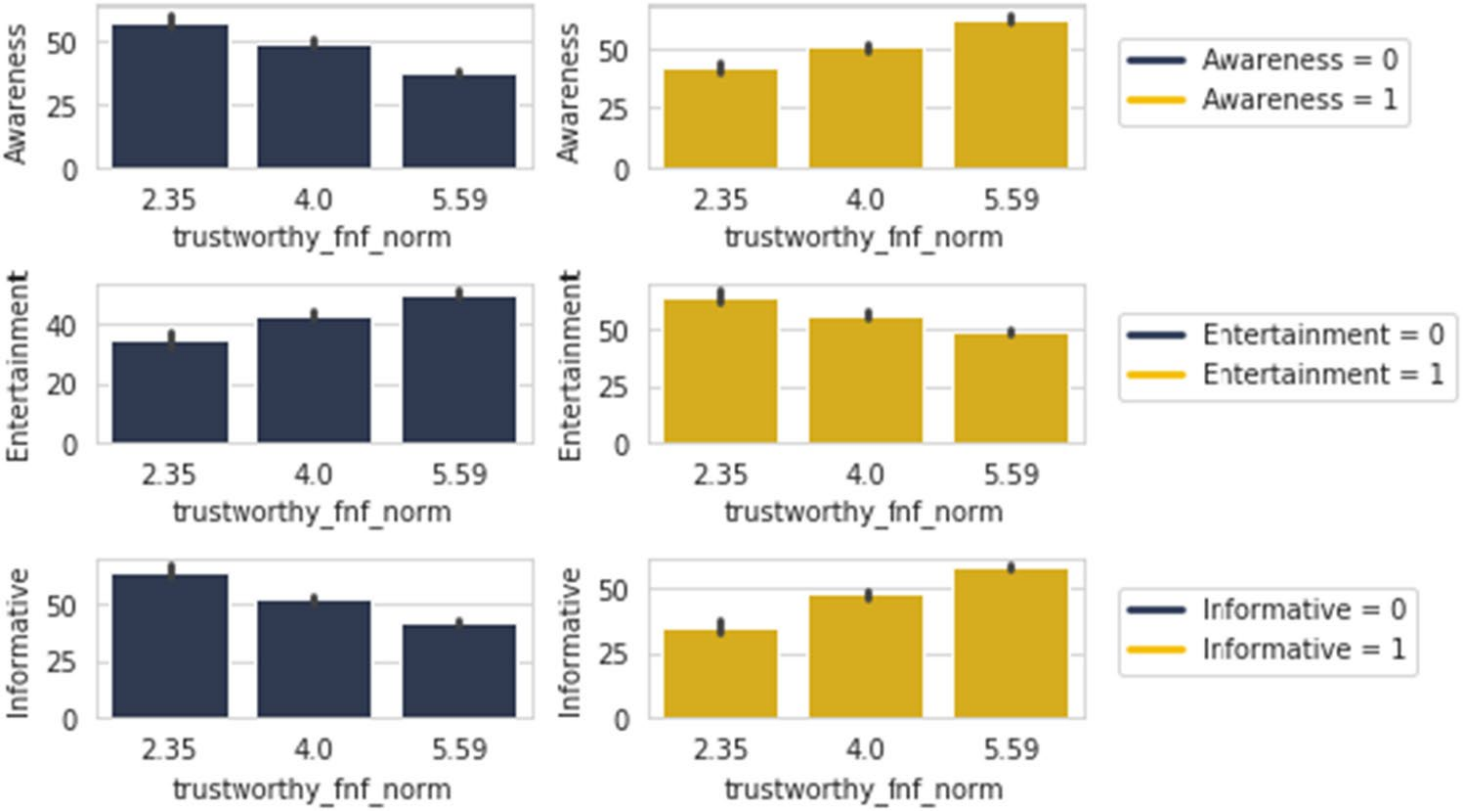
*Trustworthy FNF* with *Awareness, Entertainment* and *Informative*.

Figure 13 is showing that when *Awareness* and *Informative* contents are shared, the trust in peers and family members sharing of contents are also on the rise. Yet, while sharing *Entertainment* posts, the trust has a downward trend nature. The opposite trends are also observed in the non-sharing of these contents.

**Figure 14:**
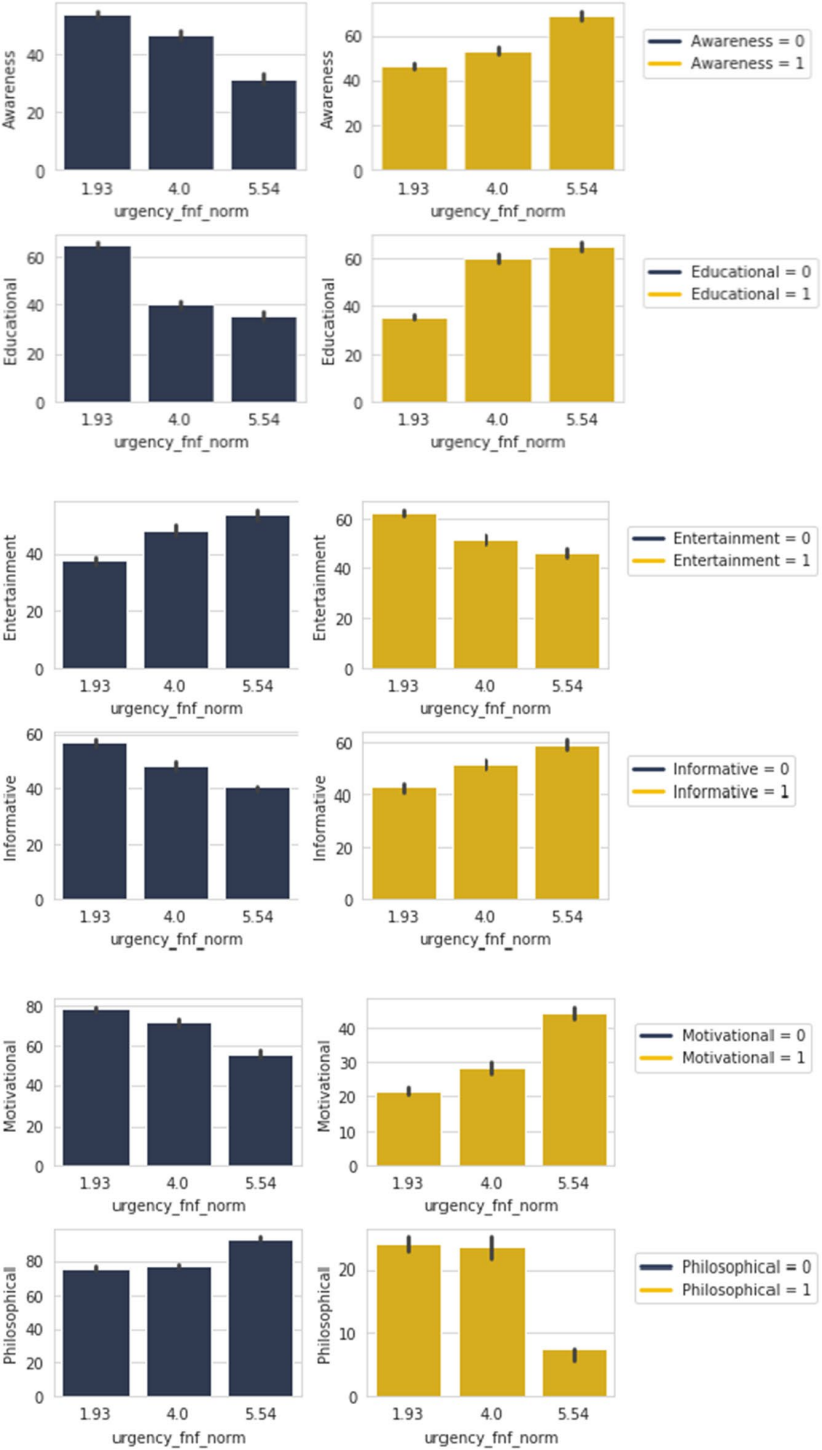
*Urgency FNF* with *Awareness, Educational, Entertainment, Informative, Motivational* and *Philosophical*.

**Table 4:** Percentage of people in the positive spectrum of *Share FNF Personal Trust* and *Trustworthy FNF* who are *Urgency FNF = 5.55* and share *Awareness, Educational, Entertainment, Informative, Motivational* and *Philosophical* content.

Urgency FNF = 5.54			
Share FNF Personal Trust			
	Maybe	Yes	Cumulative
Awareness = 1	35.1%	54.1%	89.2%
Educational = 1	34.3%	62.9%	97.2%
Entertainment = 1	32.0%	60.0%	92%
Informative = 1	34.4%	53.1%	87.5%
Motivational = 1	37.5%	58.3%	95.8%
Philosophical = 1	0.0%	100%	100%

Trustworthy FNF			
	4.00	5.59	Cumulative
Awareness = 1	5.4%	94.6%	100%
Educational = 1	11.4%	88.6%	100%
Entertainment = 1	20.0%	80.0%	100%
Informative = 1	12.5%	87.5%	100%
Motivational = 1	12.5%	87.5%	100%
Philosophical = 1	0.0%	100%	100%

*Awareness*, *Educational*, *Informative* and *Motivational* posts shared by the responders relates to the urgency in sharing posts from their peers and family are gravitating in an upward direction as seen from Fig. 14. While in the case of sharing *Entertainment* and somewhat *Philosophical* posts, the direction is on the flip side for urgency. Opposite trends are observed when the these type of posts are not shared.

Looking further into how this relationship may be aligned to features relating to personal bonds, *Share FNF Personal Trust* and *Trustworthy FNF*, we can look in to Table 4. In this table we are considering the group of people who find it urgent to share content from personally trusted human sources *Urgency FNF = 5.54* and share *Awareness, Educational, Entertainment, Informative, Motivational* and *Philosophical* content.

The table shows all of these share types, barring one, to have percentages in the range 50–63% in terms of “Yes” and a cumulative from 87 to 96% in all cases for people who would want to share our fabricated news if it came from a personally trusted human source. High percentages of around 90% is seen in terms of “Yes” with two in 80% and 100% respectively and cumulative of 100% for people who would believe in any news shared by trusted sources. This shows that people who always urgently want to share any news coming from a personally trusted human source would believe it most times which may lead to no verification.

**Figure 15:**
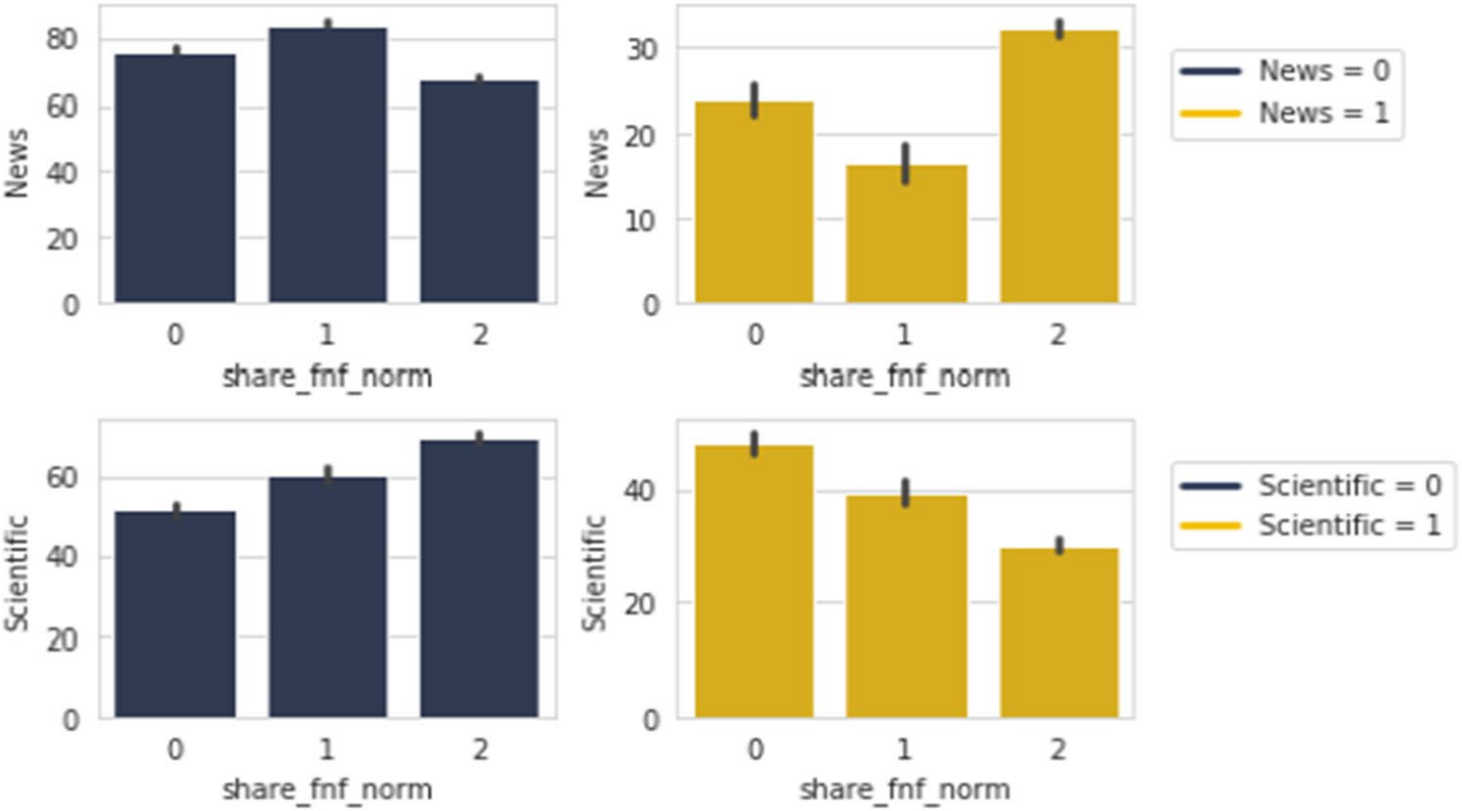
*Share FNF* with *News* and *Scientific*.

Figure 15 is showing that while sharing *Scientific* posts, the trend in sharing our supplied fabricated news has shown a dip. While in the case of *News* contents, it has taken the form of a U-shaped curve. The reverse nature is seen when among people who do not share these content types.

**Figure 16:**
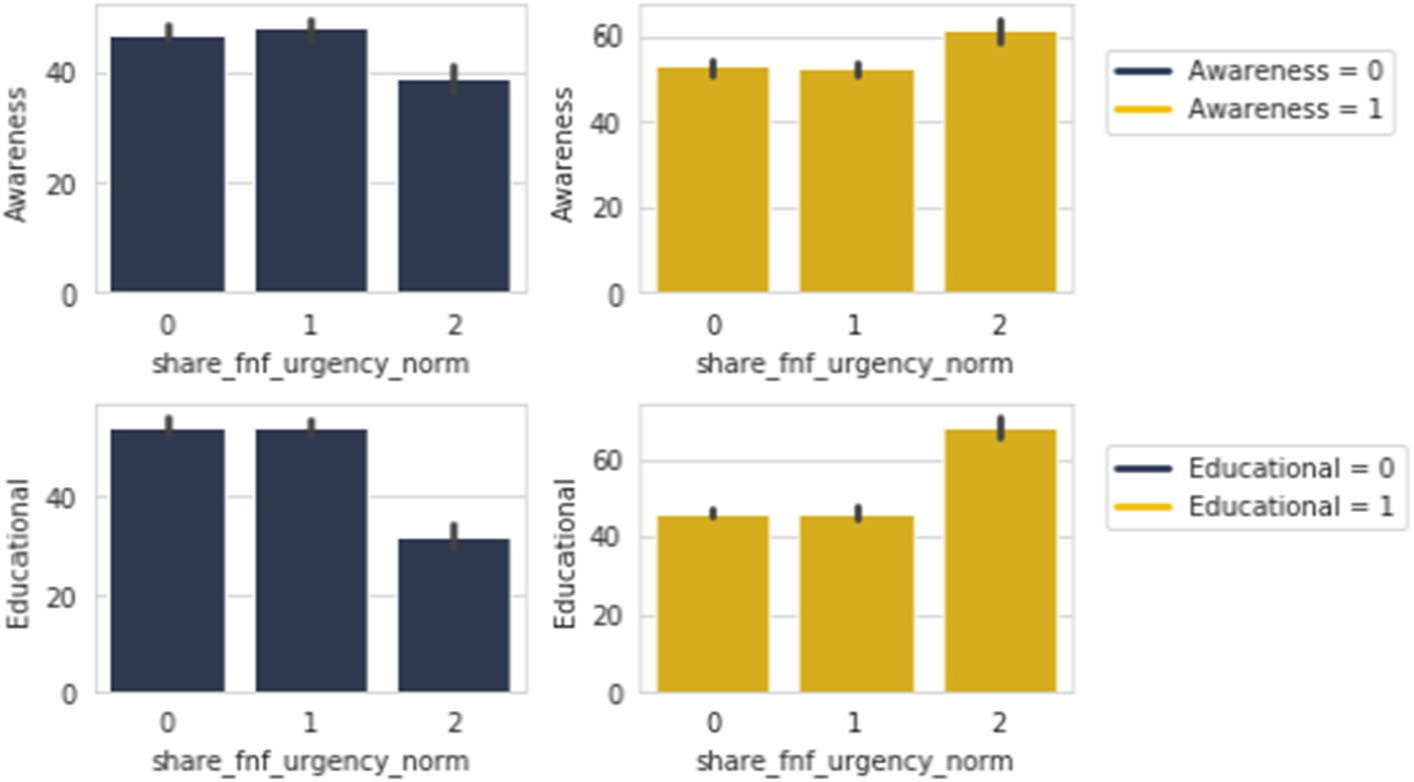
*Share FNF Urgency* with *Awareness* and *Educational*.

Figure 16 points to the trend of wanting to share our fabricated news as soon as finishing the survey, as rising among responders who share *Awareness* and *Educational* contents. The similar opposite trend is observed when these are not.

**Figure 17:**
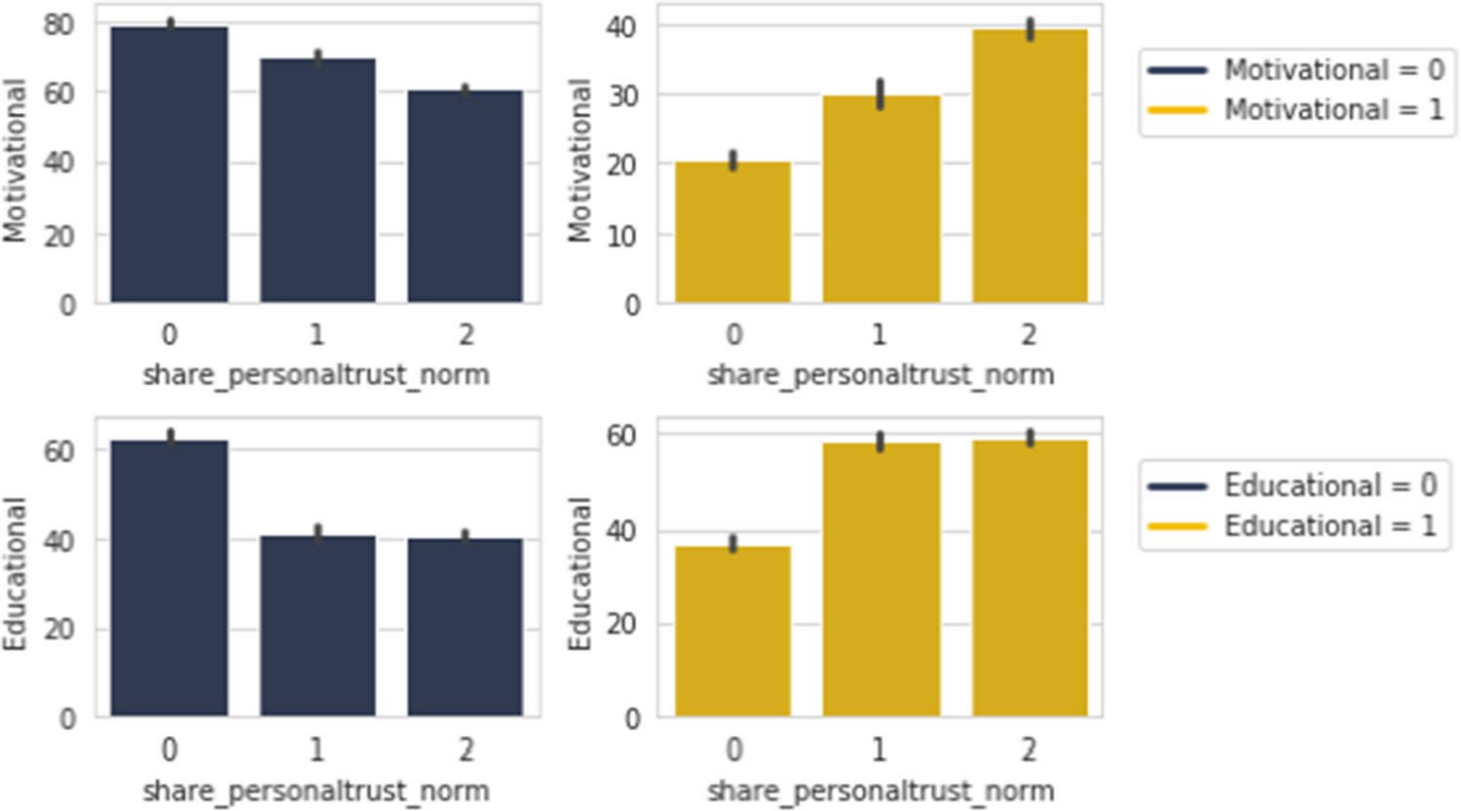
*Share FNF Personal Trust* with *Motivational* and *Educational*.

The rising trend is seen again in wanting to share our made up news among peers and friends from the group of people who share *Motivational* and *Educational* posts as shown in Fig. 17. However, people who do not share these types have shown a similar negative trend in wanting to share our fabricated news among peers, family and friends.

### Relation with different social media platforms

#### All social media platforms used

The responders were tasked with marking all the social media platforms they use daily, the list included *Facebook*, *Whatsapp*, *Instagram*, *Twitter*, *YouTube*, *TikTok*, *Snapchat*, *Reddit* and *Others*. The following are the noticeable observations made:

**Table 5:** Percentage of the number of people using *Instagram* and *WhatsApp* against the feature *Correct News FNF*.

Correct News FNF			
Instagram	0	1	2
0	56.3%	43.2%	39.4%
1	43.4%	56.8%	60.6%
WhatsApp			
0	43.8%	18.2%	16.9%
1	56.3%	81.2%	83.1%
Number of Responders	16	44	142

From Table 5, we can state that people who are more inclined in correcting their close ones or peers for false news tend to use *Instagram* and *Whatsapp* significantly more.

**Table 6:** Percentage of the number of people using *Instagram* against the feature *Share FNF*.

Share FNF			
Instagram	0	1	2
0	51.7%	35.4%	38.5%
1	48.3%	64.6%	61.5%
Number of Responders	58	48	96

For this feature in Table 6, responders who wanted or were unsure in sharing our supplied fabricated news used *Instagram* significantly more compared to responders who did not want to share.

#### Most used social media platforms

The survey takers were also tasked in marking their most used social media platform. The list included *Facebook*, *Whatsapp*, *Instagram*, *Twitter*, *YouTube*, *TikTok*, *Snapchat*, *Reddit* and *Others* as before. The following are the noteworthy observations made:

**Table 7:** Percentage of the people having *YouTube* and *Facebook* as their most used social media platform against the three values of the feature *Trustworthy FNF*.

Trustworthy FNF			
Social Media Platform	2.35	4	5.95
YouTube	45%	41.8%	26%
Facebook	45%	43%	55%
Number of Responders	40	67	95

**Table 8:** Percentage of the people having *YouTube* and *Facebook* as their most used social media platform against the three values of the feature *Urgency FNF*.

Urgency FNF			
Social Media Platform	1.39	4	5.54
YouTube	39.8%	36.7%	25.9%
Facebook	47%	50%	50%
Number of Responders	88	60	54

**Table 9:** Percentage of the people having *YouTube* and *Facebook* as their most used social media platform against the three values of the feature *Share FNF*.

Share FNF			
Social Media Platform	0	1	2
YouTube	43.1%	35.4%	30.2%
Facebook	43.1%	56.3%	49%
Number of Responders	58	48	96

**Table 10:** Percentage of the people having *YouTube* and *Facebook* as their most used social media platform against the three values of the feature *Share FNF Urgency*.

Share FNF Urgency			
Social Media Platform	0	1	2
YouTube	40.8%	30.2%	29.3%
Facebook	43.9%	57.1%	48.9%
Number of Responders	98	63	41

**Table 11:** Percentage of the people having *YouTube* and *Facebook* as their most used social media platform against the three values of the feature *Share FNF Personal Trust*.

Share FNF Personal Trust			
Social Media Platform	0	1	2
YouTube	43.6%	32.1%	28.2%
Facebook	43.6%	52.8%	52.1%
Number of Responders	78	53	71

From Tables 7, 8, 9, 10, and 11, we have been able to observe significant drops in percentages for *YouTube* whereas the changes were not much for *Facebook*. Upon observing the pattern it can be stated that with higher values of these features, responders are less likely to have *YouTube* as their most used social media platform.

## Conclusion

### Summary of key findings

By this study, we have been able to show a good amount of positive relationship between the trust of the source and the hastiness in which an piece of information can be shared skipping proper verification. It has been supported by the type of news that we had supplied and how it influenced the views of the survey takers somewhat. Supplying fabricated news has helped us in measuring these quantities in a robust way. The related use of social media platforms has also been portrayed in various methods using these features. The use of time on the internet and social media platforms has also shown what the key factors are in an individual not fact checking and spreading a suspected fake news to other parts of the larger population. Looking into the type of contents shared by the responders along with the use of specific social media platforms has helped us in taking a deeper look and profiling what type of individuals may be susceptible in contributing to the spreading of fake news whilst not verifying.

### Significance of this study

Based on our findings, we would suggest that countries such as Bangladesh in global south to put more resources in educating the population in social media safety and ethics. It is important to promote media literacy and encourage individuals to fact-check information before sharing it. This should allow to address the susceptibility one may have in taking part in this big chain of fake news spreading. Given that our target group in this study has primarily consisted of university level students who are considered to be the most proactive in social media, the findings only provide more on how awareness of fake news, their consequences and the importance of verification needs to be addressed at grassroots level. Big social media companies can also implement stricter guidelines in what kind of content they would want to show their users based on their activity and susceptibility to different kinds of information. Implementing verification mechanisms on social media platforms and targeting specific demographics or social media platforms that are more susceptible to spreading fake news can also help to curb its propagation. Further research work in placing trust ratings on different news sources on social media platforms would be of immense help and should aid in more proper monitoring of the circulation of fabricated news. We believe addressing the root causes of the spreading of fake news, individuals can become more conscious in their decision to share information, contributing to a more safe and secure cyberspace in Bangladesh, which in turn can promote a more healthier, sustainable and assured lifestyle in such countries. It is essential to take actions at multiple levels such as education, technology and targeted interventions in order to achieve this goal.

## Supplementary Information

Supplementary Figures.

## Data Availability

The datasets used and analysed during the study are available from the corresponding author. The Github respository link to the original dataset is: https://github.com/Shashwata247/Fake-News-Survey-Data.git
