# Supplementary material for: A cognitive behaviour data analysis on the use of social media in global south context focusing on Bangladesh
Source: Sci Rep. 2023 Mar 14;13:4236. doi: 10.1038/s41598-023-30125-w (PMC10013298; doi:10.1038/s41598-023-30125-w)
Supplement: Supplementary file 1 — Supplementary Figures. [file 41598_2023_30125_MOESM1_ESM.pdf]

APPENDIX

A IMAGES IN OUR SURVEY

A.1 STIMULI USED AT THE INITIAL PART OF OUR SURVEY

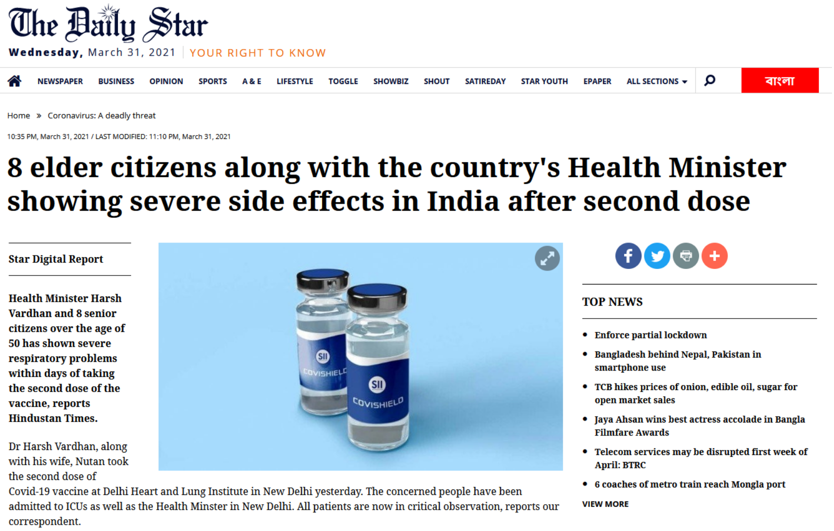
The Daily Star

Wednesday, March 31, 2021 | YOUR RIGHT TO KNOW

NEWSPAPER BUSINESS OPINION SPORTS A & E LIFESTYLE TOGGLE SHOWBIZ SHOUT SATIREDAY STAR YOUTH EPAPER ALL SECTIONS বাংলা

Home » Coronavirus: A deadly threat

10:35 PM, March 31, 2021 / LAST MODIFIED: 11:10 PM, March 31, 2021

8 elder citizens along with the country's Health Minister showing severe side effects in India after second dose

Star Digital Report

Health Minister Harsh Vardhan and 8 senior citizens over the age of 50 has shown severe respiratory problems within days of taking the second dose of the vaccine, reports Hindustan Times.

Dr Harsh Vardhan, along with his wife, Nutan took the second dose of Covid-19 vaccine at Delhi Heart and Lung Institute in New Delhi yesterday. The concerned people have been admitted to ICUs as well as the Health Minster in New Delhi. All patients are now in critical observation, reports our correspondent.

TOP NEWS

- Enforce partial lockdown
- Bangladesh behind Nepal, Pakistan in smartphone use
- TCB hikes prices of onion, edible oil, sugar for open market sales
- Jaya Ahsan wins best actress accolade in Bangla Filmfare Awards
- Telecom services may be disrupted first week of April: BTRC
- 6 coaches of metro train reach Mongla port

VIEW MORE

Fig. A.1: Fabricated news from a renowned english news outlet, The Daily Star in Bangladesh

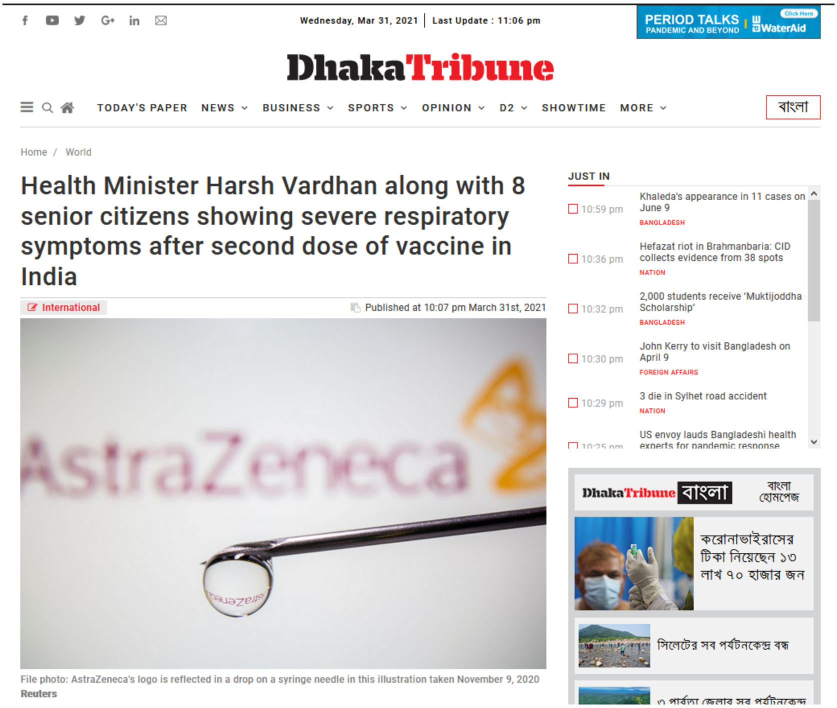

Wednesday, Mar 31, 2021 | Last Update : 11:06 pm

PERIOD TALKS PANDEMIC AND BEYOND | WaterAid

DhakaTribune

TODAY'S PAPER NEWS BUSINESS SPORTS OPINION D2 SHOWTIME MORE বাংলা

Home / World

Health Minister Harsh Vardhan along with 8 senior citizens showing severe respiratory symptoms after second dose of vaccine in India

International

Published at 10:07 pm March 31st, 2021

File photo: AstraZeneca's logo is reflected in a drop on a syringe needle in this illustration taken November 9, 2020
Reuters

JUST IN

10:59 pm Khaleda's appearance in 11 cases on June 9
BANGLADESH

10:36 pm Hefazat riot in Brahmanbaria: CID collects evidence from 38 spots
NATION

10:32 pm 2,000 students receive 'Muktijoddha Scholarship'
BANGLADESH

10:30 pm John Kerry to visit Bangladesh on April 9
FOREIGN AFFAIRS

10:29 pm 3 die in Sylhet road accident
NATION

US envoy lauds Bangladeshi health experts for pandemic response

DhakaTribune বাংলা — বাংলা হোমপেজ

করোনাভাইরাসের টিকা নিয়েছেন ১৩ লাখ ৭০ হাজার জন

সিলেটের সব পর্যটনকেন্দ্র বন্ধ

Fig. A.2: Fabricated news from another renowned english news outlet, The Dhaka Tribune in Bangladesh

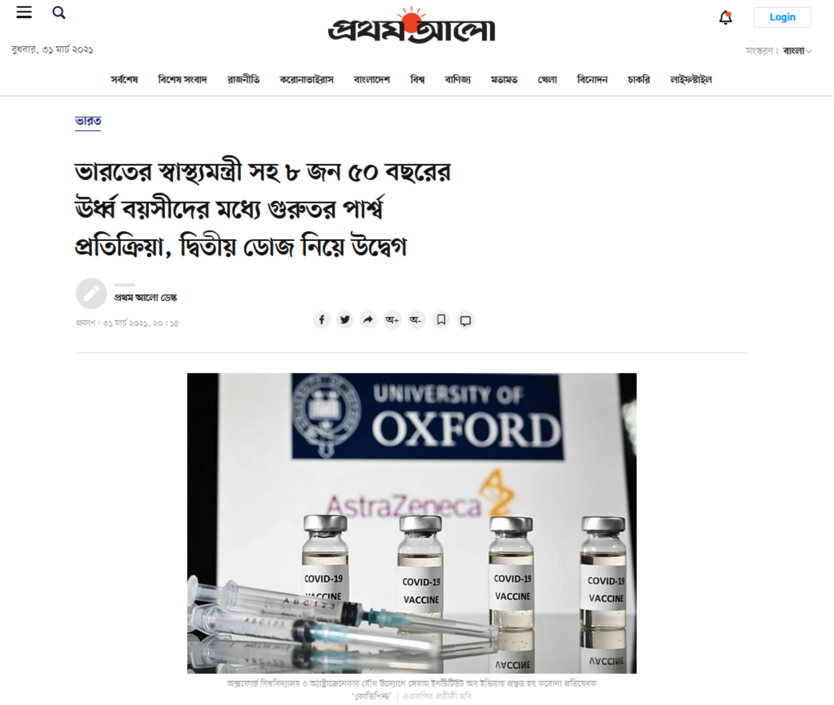
প্রথম আলো
Login
বুধবার, ৩১ মার্চ ২০২১
সংস্করণ : বাংলা
সর্বশেষ বিশেষ সংবাদ রাজনীতি করোনাভাইরাস বাংলাদেশ বিশ্ব বাণিজ্য মতামত খেলা বিনোদন চাকরি লাইফস্টাইল

ভারত

ভারতের স্বাস্থ্যমন্ত্রী সহ ৮ জন ৫০ বছরের ঊর্ধ্ব বয়সীদের মধ্যে গুরুতর পার্শ্ব প্রতিক্রিয়া, দ্বিতীয় ডোজ নিয়ে উদ্বেগ

প্রথম আলো ডেস্ক
প্রকাশ : ৩১ মার্চ ২০২১, ২০ : ১৫

অক্সফোর্ড বিশ্ববিদ্যালয় ও অ্যাস্ট্রাজেনেকার যৌথ উদ্যোগে সেরাম ইনস্টিটিউট অব ইন্ডিয়ায় প্রস্তুত হয় করোনা প্রতিষেধক ‘কোভিশিল্ড’ | এএফপির প্রতীকী ছবি

Fig. A.3: Fabricated news from a renowned bangla news outlet, Prothom Alo in Bangladesh

A.2 DECLARATION PHOTOS OF THE FABRICATION OF THE SUPPLIED NEWS

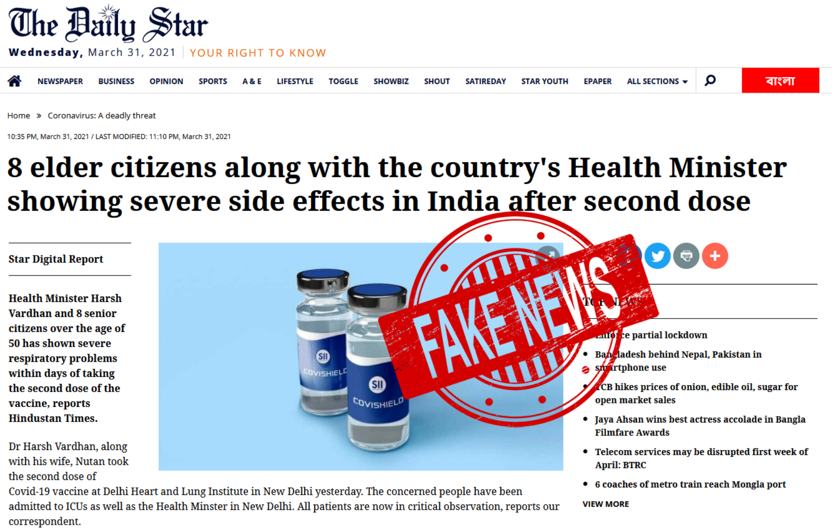
The Daily Star

Wednesday, March 31, 2021 | YOUR RIGHT TO KNOW

NEWSPAPER BUSINESS OPINION SPORTS A & E LIFESTYLE TOGGLE SHOWBIZ SHOUT SATIREDAY STAR YOUTH EPAPER ALL SECTIONS বাংলা

Home » Coronavirus: A deadly threat

10:35 PM, March 31, 2021 / LAST MODIFIED: 11:10 PM, March 31, 2021

8 elder citizens along with the country's Health Minister showing severe side effects in India after second dose

Star Digital Report

Health Minister Harsh Vardhan and 8 senior citizens over the age of 50 has shown severe respiratory problems within days of taking the second dose of the vaccine, reports Hindustan Times.

Dr Harsh Vardhan, along with his wife, Nutan took the second dose of Covid-19 vaccine at Delhi Heart and Lung Institute in New Delhi yesterday. The concerned people have been admitted to ICUs as well as the Health Minster in New Delhi. All patients are now in critical observation, reports our correspondent.

TO[illegible] NEWS

- [illegible]nforce partial lockdown
- Ba[illegible]ladesh behind Nepal, Pakistan in sm[illegible]rtphone use
- [illegible]CB hikes prices of onion, edible oil, sugar for open market sales
- Jaya Ahsan wins best actress accolade in Bangla Filmfare Awards
- Telecom services may be disrupted first week of April: BTRC
- 6 coaches of metro train reach Mongla port

VIEW MORE

Fig. A.4: Declaring The Daily Star news supplied as fabricated

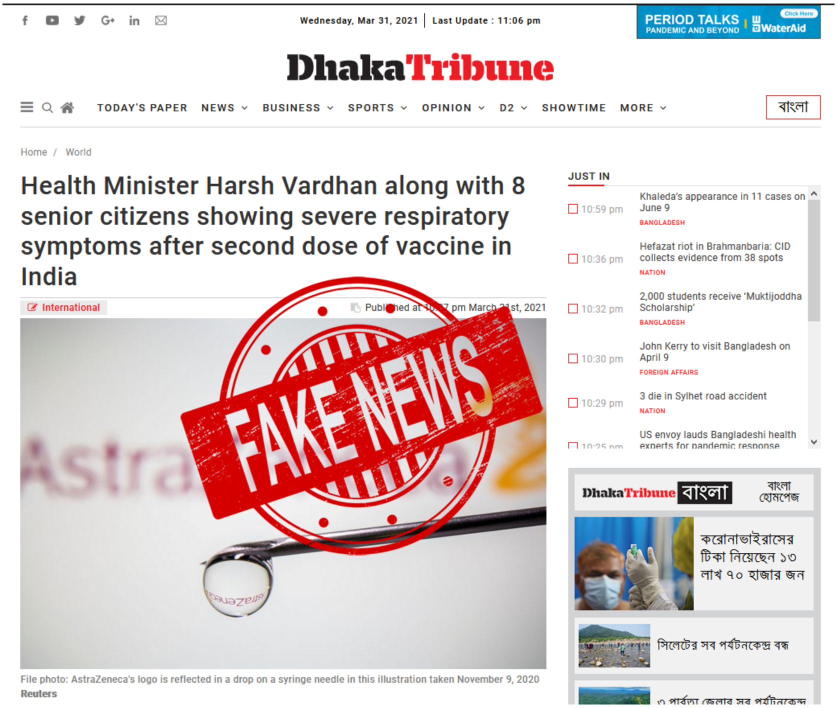

Wednesday, Mar 31, 2021 | Last Update : 11:06 pm

PERIOD TALKS PANDEMIC AND BEYOND | WaterAid | Click Here

DhakaTribune

TODAY'S PAPER NEWS BUSINESS SPORTS OPINION D2 SHOWTIME MORE বাংলা

Home / World

Health Minister Harsh Vardhan along with 8 senior citizens showing severe respiratory symptoms after second dose of vaccine in India

International — Published at 10:[illegible]7 pm March [illegible]1st, 2021

File photo: AstraZeneca's logo is reflected in a drop on a syringe needle in this illustration taken November 9, 2020
Reuters

JUST IN

- 10:59 pm — Khaleda's appearance in 11 cases on June 9 — BANGLADESH
- 10:36 pm — Hefazat riot in Brahmanbaria: CID collects evidence from 38 spots — NATION
- 10:32 pm — 2,000 students receive 'Muktijoddha Scholarship' — BANGLADESH
- 10:30 pm — John Kerry to visit Bangladesh on April 9 — FOREIGN AFFAIRS
- 10:29 pm — 3 die in Sylhet road accident — NATION
- 10:25 pm — US envoy lauds Bangladeshi health experts for pandemic response

DhakaTribune বাংলা — বাংলা হোমপেজ

- করোনাভাইরাসের টিকা নিয়েছেন ১৩ লাখ ৭০ হাজার জন
- সিলেটের সব পর্যটনকেন্দ্র বন্ধ
- ৩ পার্বত্য জেলার সব পর্যটনকেন্দ্র

Fig. A.5: Declaring The Dhaka Tribune news supplied as fabricated

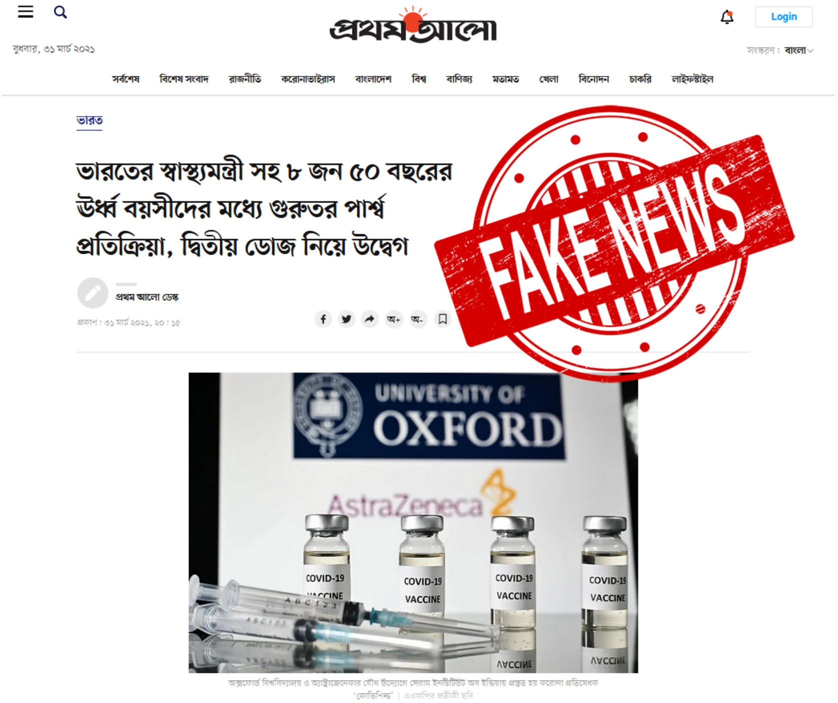

Fig. A.6: Declaring Prothom Alo news supplied as fabricated

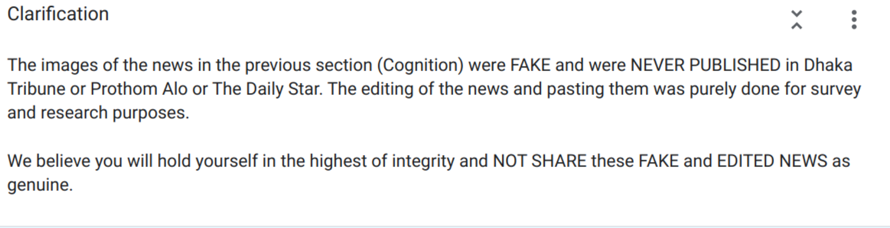
Clarification

The images of the news in the previous section (Cognition) were FAKE and were NEVER PUBLISHED in Dhaka Tribune or Prothom Alo or The Daily Star. The editing of the news and pasting them was purely done for survey and research purposes.

We believe you will hold yourself in the highest of integrity and NOT SHARE these FAKE and EDITED NEWS as genuine.

Fig. A.7: Clarification statement given at the end of our survey
